# Association of social participation and patterns with depression: analysis of data from the China health and retirement longitudinal study

**DOI:** 10.1186/s12888-025-06692-9

**Published:** 2025-04-04

**Authors:** Dongxu Chen, Sainan Duan, Jing Shi, Xiaoqin Jiang, Yida Wang

**Affiliations:** 1 Department of Anesthesiology, West China Second Hospital, Sichuan University, No. 20, Sect. 3 People South Road, Chengdu, China; 2 Key Laboratory of Birth Defects and Related Diseases of Women and Children, Sichuan University, Ministry of Education, Chengdu, China; 3 Department of Anesthesiology, The Affiliated Hospital of Guizhou Medical University, Guiyang, China; 4 Department of Anesthesiology, Chengdu Hi-Tech Zone Hospital for Women and Children, Chengdu, China; 5 Key Laboratory of BioResource and Eco-Environment of Ministry of Education, College of Life Science, Sichuan University, Chengdu, China

**Keywords:** Social participation, Depression, Trajectory, China, Major activity patterns

## Abstract

**Background:**

Considering the significance of self-reported social participation in the context of depression, patterns of social engagement may influence the onset and progression of depressive disorders. This study aimed to elucidate the relationship between social behaviors and depressive symptoms.

**Methods:**

Utilizing data from the China Health and Retirement Longitudinal Study (CHARLS) over a nine-year span, we enrolled 9415 participants without depression in 2011 and monitored them for depressive symptoms until 2020. Baseline data included ten items related to social activities. We investigated the correlation between these social activity-related items, their major patterns, and the emergence of depressive symptoms, alongside varying depressive trajectories.

**Results:**

Individual analysis of social activity-related items demonstrated significant correlations with a decreased risk of developing new depressive symptoms and adverse depressive trajectories. Pattern analysis indicated that a higher level of engagement in activities such as frequent interactions with friends (odds ratio [OR] = 0.79, 95% confidence interval [CI] 0.70–0.89), participation in diverse societal activities (OR = 0.70, CI 0.62–0.80), and a combined pattern of internet use and friend interactions (OR = 0.60, CI 0.41–0.79) was associated with a lower risk of depression onset. Compared to individuals categorized in the societal isolation group, those with a higher adherence to these activity patterns exhibited a decreased risk of developing unfavorable depressive trajectories (ORs = 0.39 to 0.83).

**Conclusions:**

Patterns of social engagement, particularly frequent interactions with friends, participation in a variety of societal activities, and the combined use of the internet with friend interactions, were inversely related to the risk of depression onset and worsening depressive trajectories. These findings emphasize that promoting social participation activities can serve as practical intervention tools to prevent depression, providing a foundation for strengthening public health policies aimed at fostering social connectivity and reducing the burden of depressive disorders.

**Supplementary Information:**

The online version contains supplementary material available at 10.1186/s12888-025-06692-9.

## Introduction

Depression is the leading contributor to the burden of mental health-related diseases and has a major source of disability globally. Approximately 280 million individuals are affected by depression, resulting in more than 47 million disability-adjusted life years in 2019 [1]. The implications of depression extend beyond its direct physical and social disabilities, as evidenced by its association with significantly elevated healthcare expenditures and a substantial reduction in the quality of life [2, 3]. Additionally, depression is intricately linked with various long-term morbidities, notably obesity [4], cardiac disease [2], substance use disorders [5], and an increased propensity for suicide attempts [5]. 

The gap between the demand for mental health services and their availability underscores a critical challenge: conventional interventions, such as cognitive behavioral therapy and pharmacotherapy, are effective in treating depression but are limited by their reliance on intensive professional resources [6]. This limitation is particularly evident in China, where a large number of individuals with depressive disorders do not seek professional help, and only 0.5% receive adequate treatment [7]. 

The effective prevention of depression necessitates interventions targeting established risk factors, among which social interaction and communication play a pivotal role. Social participation refers to individuals’ involvement in various activities that promote social interaction, including communication, collaboration, and engagement with their community. Prior research has established that these social dynamics foster mutual support, engender a sense of belonging, and significantly mitigate social isolation [8, 9], factors that collectively contribute to improved mental health and potentially prevent depression. However, the type of social participation varies across cultural contexts [10]. For instance, middle-aged and older Chinese populations often prefer activities such as mah-jong or tai chi [11]. While Western studies on voluntary work typically focus on formal involvement in non-profit organizations [12]. 

Additionally, the interplay and overlap among various modes of social participation complicate the understanding of their individual effects on depression risk. The lack of comprehensive research addressing these interactions leaves the specific impact of distinct social participation factors on depression risk largely undefined [13]. Therefore, it is crucial to analyze overall patterns of social participation to understand their role in preventing depression.

Utilizing the community-based China Health and Retirement Longitudinal Study (CHARLS), our research undertook a cohort analysis to elucidate the relationship between social participation—emphasizing its varied patterns—and the subsequent risk of new-onset depression, as well as the distinct trajectories of depression in Chinese individuals. This approach leverages CHARLS’ extensive data to provide a nuanced understanding of how specific forms of social engagement correlate with depression outcomes in this demographic.

## Methods

### Study design and participants

The foundational dataset for this investigation was sourced from the CHARLS, a longitudinal, nationwide survey designed to collect comprehensive data on middle-aged and elderly individuals, thereby facilitating research and informing policy decisions pertinent to aging in China [14]. CHARLS initially engaged participants from 150 county-level and 450 village-level units in 2011, encompassing 17,960 community-dwelling residents (Wave 1). Subsequent waves of data collection occurred through face-to-face, computer-assisted personal interviews in 2013 (Wave 2, 18455 respondents), 2015 (Wave 3, 20543 respondents), 2018 (Wave 4, 20967 respondents), and 2020 (Wave 5, 19395 respondents). The study received ethical approval from the Peking University Institutional Review Board (IRB00001052-11015), with all participants providing written informed consent [14]. Further details are available at http://charls.pku.edu.cn/.

In this study, an initial screening of 17,596 Chinese individuals was conducted. Ultimately, 9415 participants were enrolled (Figure S1). The following data were excluded from the final sample: (i) missing data on social participation or covariates from the 2011 wave, and (ii) participants who did not complete the Center for Epidemiological Studies Depression Scale (CES-D 10) in the 2011 wave and lacked at least one valid CES-D 10 score during follow-up. The basic characteristics of the full sample population are presented in Table S1.

### Assessment of social participation

In the initial wave of the CHARLS conducted in 2011, participants were queried regarding their engagement in a range of social participation activities in the preceding month. The activities encompassed: (a) Interaction with friends; (b) Participation in games such as mah-jong, chess, cards, or attendance at community clubs; (c) Provision of unpaid assistance to family, friends, or neighbors residing elsewhere; (d) Attendance at sports, social, or other club events; (e) Involvement with community-related organizations; (f) Engagement in voluntary or charity work; (g) Care for a sick or disabled adult, not cohabiting and without financial remuneration; (h) Participation in educational or training courses; (i) Engagement in stock investment; and (j) Internet usage.

Notably, internet usage and stock investment were amalgamated into a single category of social participation. This classification is based on previous research that identifies internet usage as a novel form of social engagement, particularly beneficial for older adults [15]. Additionally, due to the relatively low proportion of internet access within the Chinese population, combining the analysis helps ensure a sufficiently large subgroup for study. Respondents affirming participation in any of the aforementioned activities were further asked to specify the frequency of their involvement (almost daily, almost every week, or irregularly). Comprehensive information about the original survey questions and the variables employed in this analysis is accessible in the online supplement.

### Identification of social participation patterns

In light of the high occurrence events of these society participation (Figure S2), we employed a two-step methodology to delineate distinct clusters of patients characterized by varying patterns of societal engagement [16]. Initially, we utilized Multiple Correspondence Analysis (MCA) – a principal component technique – to summarize and visually represent a data matrix containing various categorical dependent variables. For this purpose, categorical variables were converted into dummy variables (i.e., assigned values of 0 or 1). Subsequently, the MCA transformed these dummy variables into multi-dimensional [17]. Euclidean coordinates, subsequently generating a number of components. We opted to retain all components that cumulatively accounted for 100% of the variance in the dataset, due to no obvious inflection point was found in elbow figure.

**Fig. 1:**
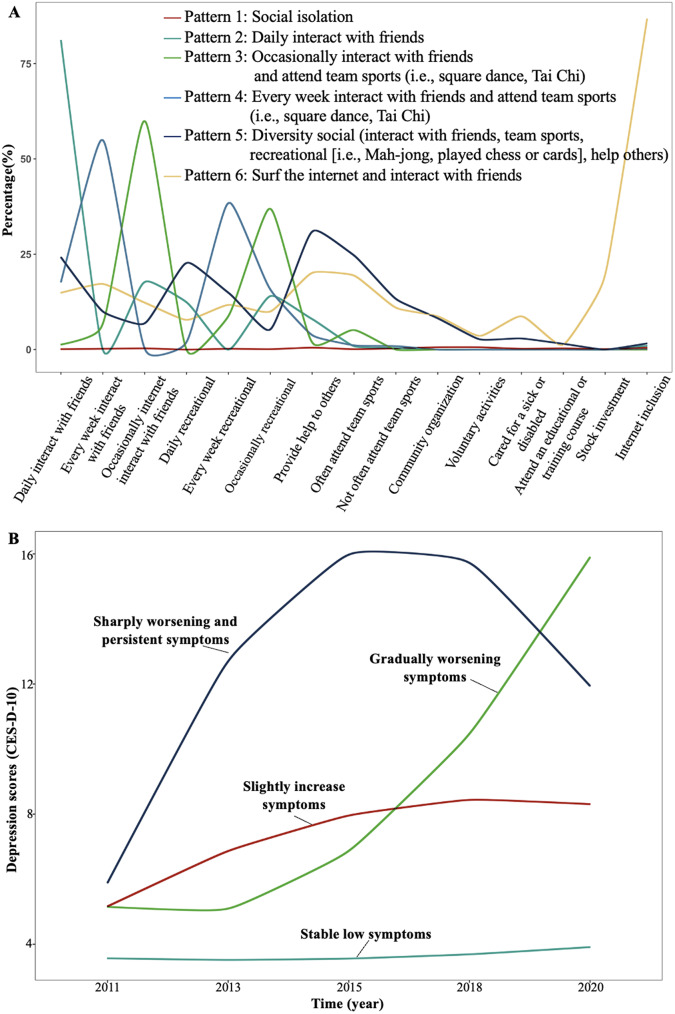
(**A**). Characteristics of the identified preoperative psychological patterns. (**B**). Trajectories of depressive symptoms. Note: (**A**) The incidence of events for ten society activities for the identified six patterns, identified by the *k-means* clustering algorithm. A higher proportion indicated a higher level of engagement with the corresponding activity pattern. The X-axis information is from the original questionnaires ID DA056 and DA057 and more details are presented in supplementary

The second phase of our analysis involved applying the *k-means* clustering algorithm to these derived components, with the aim of identifying distinct groups of participants based on their patterns of societal engagement [16]. The optimal number of clusters was determined using the elbow method [6]. This process culminated in the identification of six unique patterns of societal participation. Each cluster was labeled according to its most prominent societal participation feature, based on the variables with the highest loading values (Fig. 1 and Figure S3). These patterns were categorized as ‘social isolation’, ‘daily interact with friends’, ‘occasionally ‘and ‘every week’ interact with friends and attend team sports’, ‘diversity social’, as well as ‘surf the internet and interact with friends’, each reflecting a distinctive mode of societal engagement (Fig. 1 and Supplementary Table S2).

### Ascertainment of new onset depression and depression trajectories

The 10-item CES-D 10 was used to examine the depressive symptoms. Participants were questioned about their experiences of positive feelings, negative emotions, and somatic symptoms during the preceding week. The CES-D 10 employs a four-point scale for responses, ranging from 0 to 3. The aggregate score rangs from 0 to 30, with higher scores indicating more severe depressive symptoms. The CES-D 10, which has demonstrated good internal reliability (Cronbach’s alpha = 0.815) in prior studies, is a validated instrument for this purpose [18]. Numerous studies have identified a cutoff point of 10 as effective for recognizing clinically significant depression [19, 20]. Consequently, we adopted a cutoff point of 10 to delineate the prevalence of depression. New onset depressive symptoms were determined in individuals exhibiting signs of depression in follow-up assessments from 2012 to 2020 (wave 2 to wave 5).

Beyond the singular application of CES-D 10 data at follow-up, we implemented latent class trajectory models to discern distinct depressive symptom trajectories over a 9-year span. This analysis utilized the CES-D 10 scores recorded in 2013 (wave 2), 2015 (wave 3), 2018 (wave 4), and 2020 (wave 5). The selection of the best-fitting number of trajectories was based on the minimum Bayesian information criterion. This approach identified four distinct depressive symptom trajectories: (1) consistently low depressive symptoms (depicted in cyan, *n* = 4852 [51.5%]); (2) gradually worsening symptoms trajectory (in green, *n* = 623 [6.6%]); (3) slightly increasing trajectory (in red, *n* = 2920 [31.0%]), and (4) sharply worsening and persistent trajectories (in blue, *n* = 1020 [10.8%]) (as detailed in Fig. 1 and Supplementary Table S3). The characteristics associated with each depressive symptom trajectory are documented in Supplementary Table S4.

### Covariates

Covariates for this study were determined through extensive reviews of existing literature. Data on various demographic factors such as sex, age, and body mass index (BMI), alongside socioeconomic factors including marital status, education level, current employment status, and type of residence, were collected. Additionally, lifestyle aspects like alcohol consumption and smoking status were taken into account. The presence of somatic disease, baseline depressive symptoms, and functional status were also considered as important covariates. These data were gathered from the basic information questionnaire administered in the first wave of the study.

### Statistical analysis

We meticulously detailed the baseline characteristics of all study participants, categorizing them based on the presence or absence of new onset depressive symptoms. Directed Acyclic Graphs (DAGs) were employed to elucidate the potential roles of various covariates in the relationships under study, aligning with the hypothesized pathways (as illustrated in Supplementary Figure S4).

Subsequently, we analyzed the connections between individual social participation activities (encompassing all categorical items related to social engagement) and major participation patterns with the new onset of depressive symptoms. Additionally, we examined the correlation with different depressive symptom trajectories. These relationships were quantified using odds ratios (ORs) with 95% confidence intervals (CIs), derived from comprehensive logistic or multinomial regression models that were fully adjusted for potential confounders.

To explore the modification effects of age, sex, education level, and type of residence on the studied associations, we conducted subgroup analyses. These analyses were stratified by age groups (< 50 years, 51–59 years, or ≥ 60 years), sex (male or female), education level (lower, upper secondary and vocational training, or tertiary), and type of residence (rural, urban, or unknown).

In our sensitivity analyses, we tested the robustness of our findings against the basic depressive symptom data. This was achieved by repeating the primary analysis, excluding participants who lacked baseline depressive symptom information. A two-sided *P*-value of less than 0.05 was considered to indicate statistical significance.

For our statistical analyses, we utilized Python (version 3.8) for k-modes-based cluster analysis and R (version 4.1.1) for various tasks. These tasks included association analysis (employing the ‘nnet’ package), cluster analysis using ‘cluster’ package, MCA using the ‘FactoMineR’ package.

## Results

The mean age at baseline was 58 years, with males constituting 51.3% of the sample (4831/9415; Table S1). Among the 9415 participants, a predominant portion resided in rural areas (46.5%), exhibited lower educational attainment (84.7%), and had partners (90.9%). The majority abstained from alcohol consumption (64.1%) and smoking (59.5%), and reported conditions such as hypertension (22.0%), digestive diseases (18.6%), and arthritis or rheumatism (27.5%). We found that individuals with new onset of depression were more frequent in females (55.9% versus 42.5%), among those with lower educational levels (89.9% versus 80.3%), and to be more prone to somatic diseases and functional dependencies (Table S1). Additionally, several covariates were significantly correlated with varied patterns of societal engagement (Table S2).

**Fig. 2:**
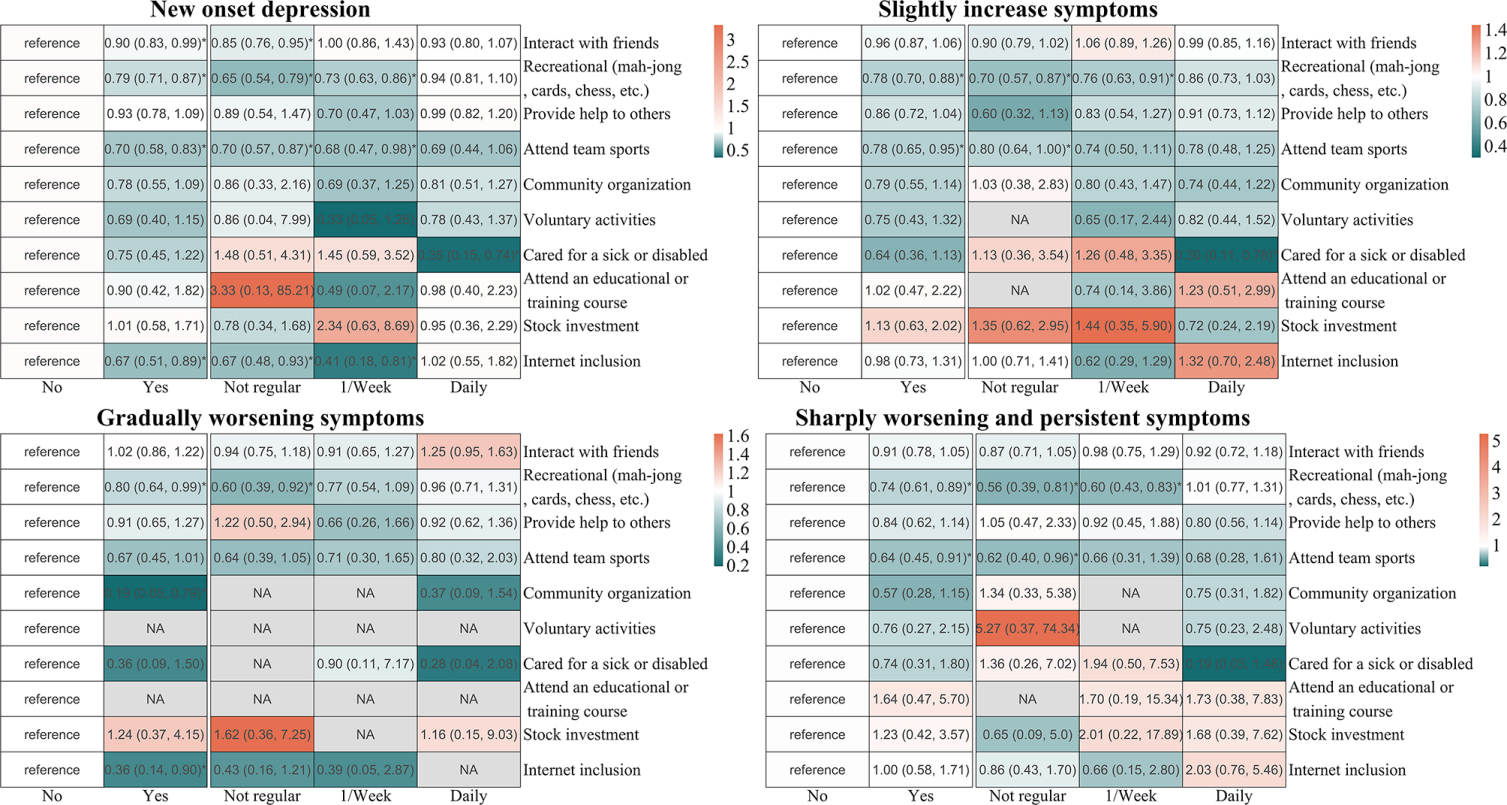
Associations between different types of social activity and depressive onset, as well as depressive trajectories. The color of the circle represents the magnitude of odds ratios (ORs), derived from fully adjusted models (adjusted for age, gender, body mass index, smoking status, drinking status, residency, educational attainment, marital status, somatic disease, baseline depressive score, and functional dependency). The variable indicating the participation (yes or no) was obtained through reclassification of the original items that were measured ordinally in the questionnaire. ‘NA’ means that there is no corresponding population under the category of this item

**Fig. 3:**
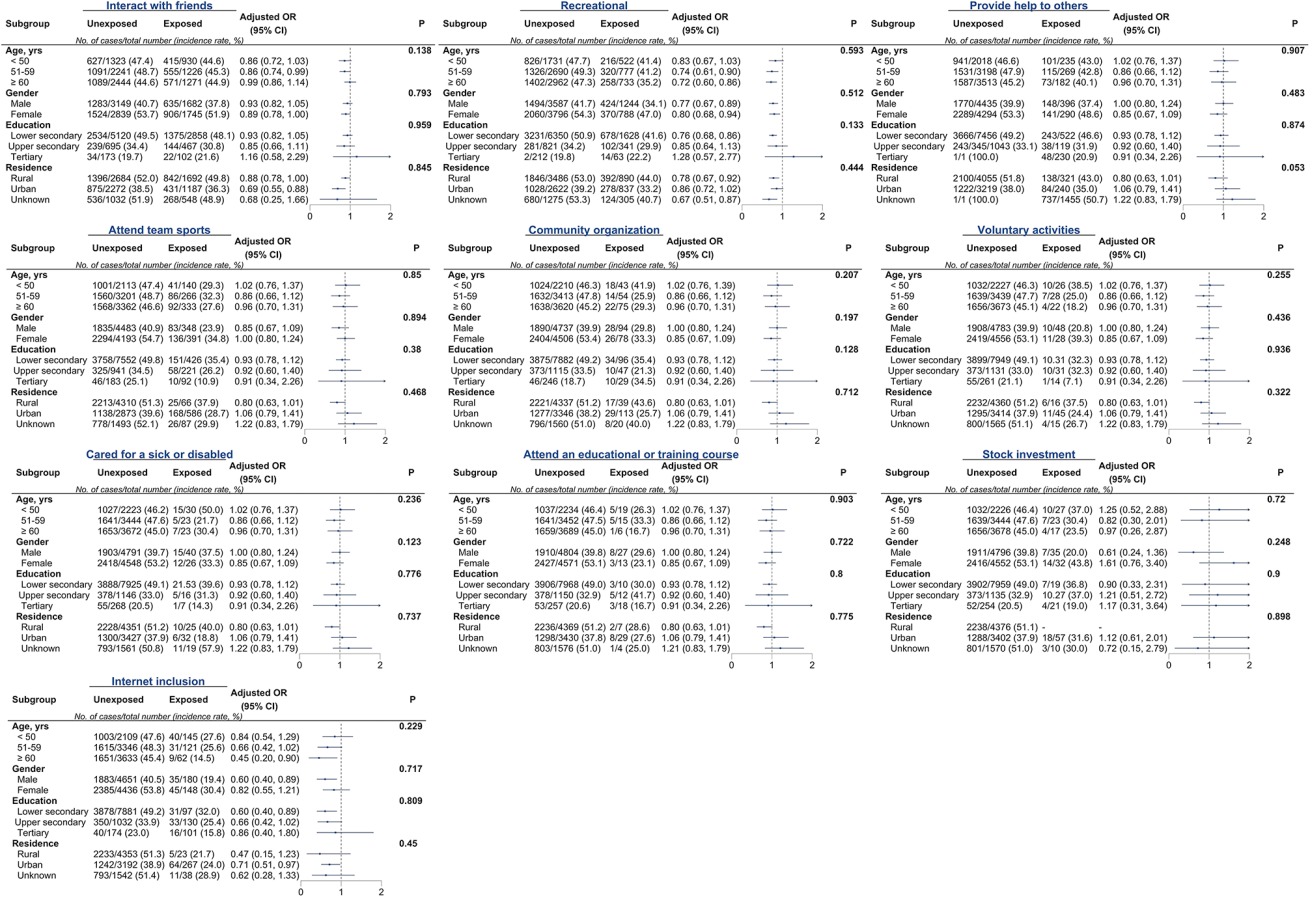
Associations between different social participation items and specific subtypes of new onset depression. The color of the circle represents the magnitude of odds ratios (ORs), derived from fully adjusted models (adjusted for age, gender, body mass index, smoking status, drinking status, residency, educational attainment, marital status, somatic disease, baseline depressive score, and functional dependency)

Figure 2 depicts the immediate relationships between depressive symptoms and the diversity, frequency, and types of societal engagement, post-adjustment for confounders. Notably, social interactions with friends (OR 0.90, 95% CI 0.80 to 0.99), participation in recreational activities (e.g., mah-jong, cards, chess) (OR 0.79, 95% CI 0.71 to 0.87), involvement in team sports (e.g., square dance, Tai Chi) (OR 0.70, 95% CI 0.58 to 0.83), and internet use (OR 0.67, 95% CI 0.51 to 0.89) demonstrated significant associations with reduced depressive symptoms. Subgroup analyses confirmed consistent associations across age, gender, education, and residency (Fig. 3).

However, an evaluation based on the frequency of social engagement yielded mixed and inconsistent findings. For instance, daily participation in team sports was not significantly associated with better depressive symptoms (OR 0.69, 95% CI 0.44 to 1.06), whereas sporadic involvement in team activities significantly associated with lower new-onset depression (OR 0.70, 95% CI 0.57 to 0.87). This trend was similarly observed in interactions with friends, internet usage, and recreational activities (Fig. 2 and Table S5). In the trajectory analysis of depressive symptoms, engagement in recreational activities, community organization, and internet use were linked to a decreased risk of progressively worsening depressive symptoms (ORs ranging from 0.19 to 0.80). Moreover, engagement in recreational activities and team sports was associated with reduced risk of experiencing sharp worsening and persistent depressive trajectories (Fig. 2 and Table S6).

**Fig. 4:**
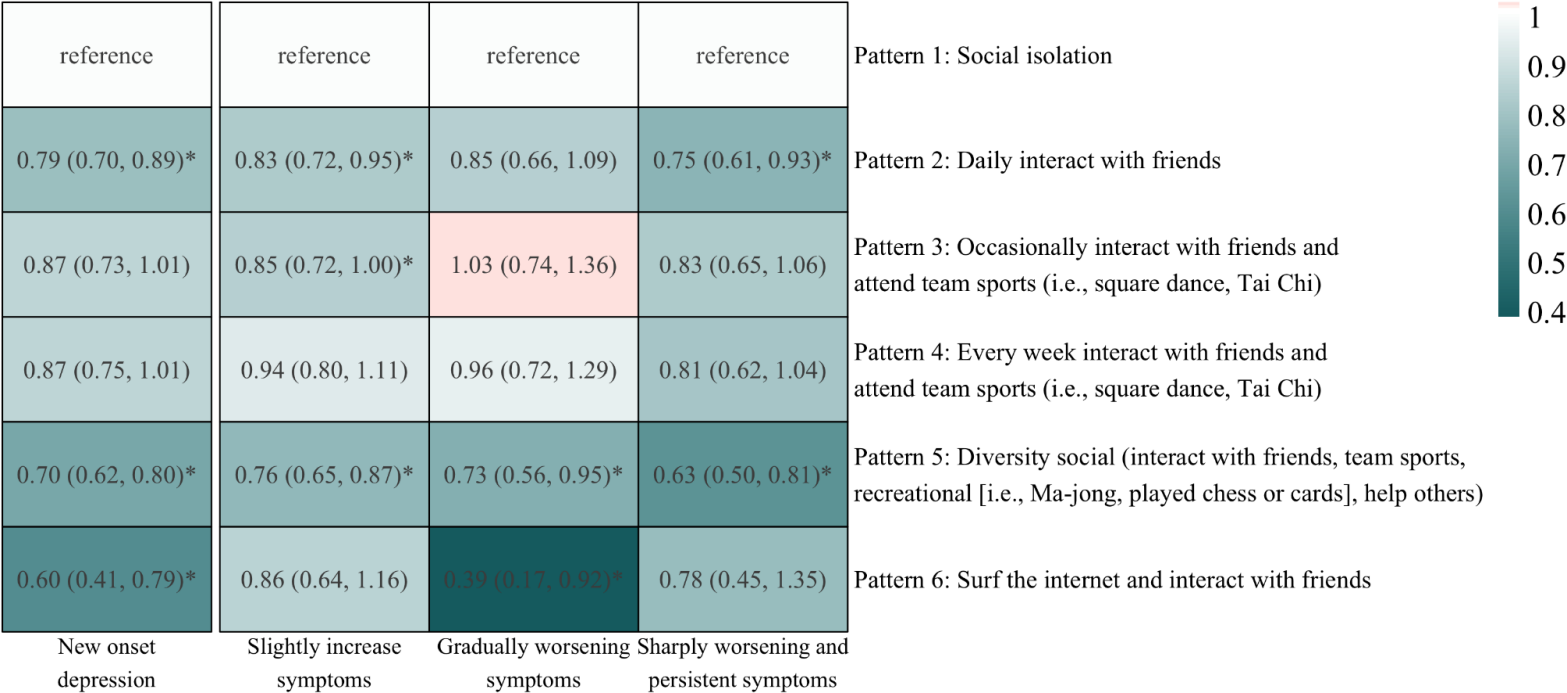
Association between patterns of society activity and depressive onset, as well as depressive trajectories. The color of the circle represents the magnitude of odds ratios (ORs), derived from fully adjusted models (adjusted for age, gender, body mass index, smoking status, drinking status, residency, educational attainment, marital status, somatic disease, baseline depressive score, and functional dependency). The variable indicating the participation (yes or no) was obtained through reclassification of the original items that were measured ordinally in the questionnaire

In examining the major patterns of societal participation, findings indicated that, compared to individuals in the societal isolation group, there was a notable reduction in the risk of new-onset depression among those who interacted daily with friends (fully adjusted OR = 0.79, 95% CI 0.70 to 0.89; Fig. 4), engaged in diverse societal activities (OR = 0.70, 95% CI 0.62 to 0.80), and combined internet use with friend interactions (OR = 0.60, 95% CI 0.41 to 0.79; Fig. 4 and Table S5). Subgroup analyses showed no variation in the impact of societal participation patterns on the incidence of new-onset depression across predefined variables (Table 1). Furthermore, daily interactions with friends pattern were associated with a declined risk of slightly increases (OR = 0.83, 95% CI 0.72 to 0.95) or sharply worsening and persistent in depressive trajectories (OR = 0.75, 95% CI 0.61 to 0.93). Similarly, diversity in societal engagement have elevated risk of experiencing unfavorable depressive trajectories, including slightly increases (OR = 0.76, 95% CI 0.65 to 0.87), gradually worsening (OR = 0.73, 95% CI 0.56 to 0.95), and sharply worsening and persistent deterioration (OR = 0.63, 95% CI 0.50 to 0.81). However, individuals with access to the internet and who interacted with friends exhibited a lower risk of gradual deterioration of the depressive trajectory (OR = 0.39, 95% CI 0.17 to 0.92).

**Table 1:** Associations between major social participation pattern and specific subtypes of new onset depression

Subgroups	No. of cases / total number (incidence rate, %)		Adjusted OR (95% CI)	No. of cases / total number (incidence rate, %)	Adjusted OR (95% CI)	No. of cases / total number (incidence rate, %)	Adjusted OR (95% CI)	No. of cases / total number (incidence rate, %)	Adjusted OR (95% CI)	No. of cases / total number (incidence rate, %)	Adjusted OR (95% CI)	*P* for interaction
	Pattern 1	Pattern 2		Pattern 3		Pattern 4		Pattern 5		Pattern 6		
**Age**,** yrs**												0.544
< 50	488/968 (50.4)	155/339 (45.7)	0.72 (0.55, 0.94)	116/258 (45.0)	0.78 (0.58, 1.04)	104/241 (43.2)	0.73 (0.54, 0.99)	137/318 (43.1)	0.89 (0.68, 1.16)	42/129 (32.6)	0.91 (0.58, 1.42)	
51–59	863/1655 (52.1)	240/500 (48.0)	0.78 (0.64. 0.97)	168/362 (46.4)	0.84 (0.66, 1.07)	155/339 (45.7)	0.83 (0.65, 1.06)	189/497 (38.0)	0.67 (0.54, 0.83)	31/114 (27.2)	0.55 (0.34, 0.85)	
≥ 60	888/1806 (49.2)	241/545 (44.2)	0.82 (0.67, 1.00)	154/325 (47.4)	0.98 (0.76, 1.25)	167/342 (48.8)	1.06 (0.83, 1.34)	202/611 (33.1)	0.67 (0.55, 0.82)	8/66 (12.1)	0.29 (0.12, 0.60)	
**Gender**												0.773
Male	994/2251 (44.2)	239/612 (39.1)	0.84 (0.70, 1.01)	200/515 (38.8)	0.82 (0.67, 1.00)	204/517 (39.5)	0.87 (0.71, 1.06)	247/767 (32.2)	0.71 (0.59, 0.85)	34/169 (20.1)	0.49 (0.32, 0.73)	
Female	1245/2178 (57.2)	397/772 (51.4)	0.74 (0.63, 0.88)	238/430 (55.3)	0.93 (0.75, 1.15)	222/405 (54.8)	0.89 (0.71, 1.11)	281/659 (42.6)	0.69 (0.57, 0.83)	47/140 (33.6)	0.79 (0.53, 1.18)	
**Education**												0.257
Lower secondary	2059/3955 (51.2)	601/1264 (47.5)	0.80 (0.70, 0.92)	391/794 (49.2)	0.90 (0.76, 1.05)	385/795 (48.4)	0.89 (0.76, 1.04)	437/1067 (41.0)	0.72 (0.62, 0.82)	36/103 (35.0)	0.64 (0.41, 0.97)	
Upper secondary and vocational training	162/410 (39.5)	31/107 (29.0)	0.59 (0.36, 0.95)	45/138 (32.6)	0.72 (0.47, 1.09)	36/110 (32.7)	0.73 (0.46, 1.15)	78/279 (28.0)	0.65 (0.46, 0.91)	31/118 (26.3)	0.52 (0.31, 0.84)	
Tertiary	18/64 (28.1)	4/13 (30.8)	0.45 (0.08, 2.15)	2/13 (15.4)	0.28 (0.03, 1.69)	5/17 (29.4)	1.69 (0.42, 6.30)	13/80 (16.3)	0.45 (0.18, 1.14)	14/88 (15.9)	0.53 (0.18, 1.14)	
**Residence**												0.910
Rural	1169/2158 (54.2)	383/767 (49.9)	0.80 (0.68, 0.95)	245/488 (50.2)	0.89 (0.73, 1.09)	228/464 (49.1)	0.83 (0.68, 1.02)	207/468 (44.2)	0.74 (0.60, 0.91)	6/31 (19.4)	0.34 (0.12, 0.80)	
Urban	632/1477 (42.8)	152/412 (36.9)	0.77 (0.61, 0.97)	128/309 (41.4)	0.97 (0.75, 1.26)	113/285 (39.6)	1.00 (0.76, 1.31)	223/743 (30.0)	0.67 (0.55, 0.82)	58/233 (24.9)	0.63 (0.44, 0.88)	
Unknown	438/794 (55.2)	101/205 (49.3)	0.77 (0.56, 1.07)	65/148 (43.9)	0.66 (0.46, 0.95)	85/173 (49.1)	0.82 (0.58, 1.15)	98/215 (45.6)	0.77 (0.56, 1.05)	17/45 (37.8)	0.74 (0.37, 1.45)	

OR, odds ratio; CI, confidence interval

Multinomial logistic regression adjusted for confounding clarified by Directed Acyclic Graphs was used to estimate ORs and 95% CIs

Sensitivity analyses, which excluded individuals with missing baseline depressive symptom status, confirmed that these risk estimates remained substantially unchanged (Figure S5-S6 and Tables S7-S8). Only 1.6% of individuals in this cohort received treatment for depression (Table S9). It is important to note that the proportion of individuals with adverse depressive trajectories who received treatment was higher than that of individuals in other trajectories grouping.

## Discussion

The findings from a community-based cohort study, encompassing over 9000 participants across a nine-year follow-up period, revealed that individual analysis of certain social activities—such as interactions with friends, engagement in recreational activities, participation in team sports, and internet usage—were linked to a diminished risk of developing new-onset depression or experiencing an adverse depression trajectory. Nonetheless, the analysis of social frequency yielded several results that were challenging to interpret and marked by inconsistencies. Conversely, the analysis of combined effects presented more uniform and plausible outcomes, demonstrating that daily interactions with friends, participation in a diverse range of social activities, and predominant internet access patterns were significantly associated with a lower risk of new-onset depression and negative depression trajectory.

Consist with our findings, a plethora of studies have elucidated the correlation between societal engagement and depression [9, 13, 21]. Wang and colleagues identified a decreased risk of depression among individuals who regularly interacted with friends, frequently played mah-jong, and participated in sports or social clubs. However, they observed no significant relationship between depressive symptoms and engagement in voluntary activities, community organizations, or internet usage [13]. Similar outcomes have been reported in research focusing on social isolation [9, 21]. Nonetheless, contrasting results have emerged from certain survey-based investigations [22]. Perlis et al., who examined 8045 participants, found that among those initially free of depressive symptoms, the use of social media was linked to a higher probability (ORs ranging from 1.39 to 1.53) of experiencing an increase in depressive symptoms [22]. Furthermore, in the present study, while engagement in recreational activities (e.g., playing mah-jong) or team sports (e.g., square dancing, Tai Chi) was associated with a reduced depression risk, this protective effect did not amplify with increased frequency of these activities, leading to more equivocal conclusions. This may be attributed to the reduced sample size when the population is further subdivided, resulting in insufficient statistical power. For instance, only 12% of individuals with daily contact with friends (379/3200) use the Internet, and only 5% (17/319) engage in this activity daily, while just 4% (30/720) participate in group activities on a daily basis. These findings underscore the importance of conducting a pattern analysis, such as cluster analysis, which considers all social activities collectively rather than treating them as separate categories. In this joint analysis, the ORs for new-onset depression in the Pattern 3 (frequency: occasional) group was 0.87, in Pattern 2 (frequency: daily) it was 0.79, and in Pattern 5 (diversity social) it decreased to 0.70. These results suggest that higher frequency of social activity is associated with a further reduction in depressive symptoms. Besides, it is important to note that the social activities data used in this study were collected over a decade ago (2011 wave), during which the role of the internet in social participation has undergone substantial changes. Since 2011, there has been a significant increase in internet access and usage in China, with the rise of social media platforms, online communication tools, and the broader digitalization of daily life likely altering how individuals engage in social activities through the internet. However, despite global trends indicating a marked shift in internet usage, CHARLS data revealed that internet usage remained relatively stable in China, only slightly fluctuating from 24.4% in 2011 to 24.2% in 2020. This suggests that, while internet use patterns have evolved globally, the extent of this change within the study population remains limited. As a result, the precise nature and extent of the relationship between social participation and depression risk continue to be debated. There remains a lack of consensus on the specific type or level of social participation that is inversely associated with depression risk.

While previous research has endeavored to identify the most beneficial forms of social engagement [13, 23, 24], the collective impact of various social activities, which often operate as a complex set within individuals, has scarcely been examined. In this vein, our application of *k-means* clustering to delineate major patterns of social participation was innovative, highlighting the intricate nature of concurrent social activities. For the first time, our analysis focused on patterns, revealing the meaningful combination patterns of social engagement on depression. These insights underscore the significance of employing a multifaceted and pattern-oriented approach to identify high-risk populations, potentially enabling tailored interventions for depression management. Our findings not only augment the existing body of research but also offer crucial insights into the dynamics between depression and social activity. On one level, this discovery illustrates the influence of Chinese cultural practices on individual behaviors and their links to mental health. On another, it underscores the critical need for continued research aimed at informing targeted policies to enhance mental well-being.

Our research holds significant implications for mental illness prevention, specifically in averting the onset of depression or its further progression, and in improving the quality of life among older adults from both public health and social policy perspectives. Our study highlights that internet usage remains disproportionately low among these individuals. To mitigate this disparity and address the unequal distribution of healthcare resources, the Chinese government has introduced the “Integrated Medical Services and Elderly Care” (IMSEC) initiative, which encompasses several models, including Home IMSEC, Community IMSEC, Institutional IMSEC, and Internet Plus IMSEC [25]. A comparable policy has been proposed by the Japanese government [26]. However, our analysis of patterns indicates that relying solely on one approach may be insufficient. A more diversified and multifaceted strategy is necessary to achieve meaningful and sustainable outcomes in improving social participation and healthcare accessibility. Such as, the Act-Belong-Commit (ABC) mental health promotion campaign, which originated in Western Australia and has since been implemented globally, stands as a model of excellence [9]. The campaign encourages individuals to adopt behaviors conducive to mental well-being, including maintaining social activity, cultivating social relationships, and engaging in meaningful and purposeful endeavors, while also supporting community or team organizations in facilitating opportunities for such health-promoting engagements [27]. 

### Strengths and limitations

The present study has several advantages. First, the utilization of a sizable and uniform patient cohort, enriched with comprehensive data on clinical details, social engagement, and depressive symptom assessments collected both independently and prospectively, substantially minimizes the risk of information bias. This approach allows for the detailed exploration of relevant associations across diverse subgroup strategies. Second, our pioneering use of clustering analyses to discern significant patterns of societal engagement (e.g., k-means analysis) and to identify longitudinal changes in depressive symptoms (e.g., latent class trajectory analysis) represents an innovative contribution to the field. Importantly, the former analysis facilitates a comprehensive examination of an individual’s societal participation as a cohesive unit, ensuring that the exposed and reference groups in our analyses are both realistic and mutually exclusive, thereby yielding meaningful comparisons [28]. Moreover, the latter analysis sheds light on categorizing individuals with poor depression-related outcomes, thereby enhancing the clinical significance of our findings.

However, this study is not without its limitations. First, a limitation of this study is the exclusion of participants with missing data, which may introduce bias and this should be considered when interpreting the findings. Then, changes in social participation may coincide with alterations in other variables linked to depressive symptoms [29]. For instance, older individuals might reduce their involvement in team or recreational activities following significant life events, such as the birth of a grandchild. Additionally, the nature and frequency of social activity participation may evolve over time, necessitating a cautious interpretation of our results. The impact of these variables on our findings is complex and unpredictable, as they could potentially increase rather than decrease depression levels, possibly leading to an underestimation of the relationship between engagement in certain activities and depression. Another limitation concerns the potential for reverse causality, while we observed that participation in team or recreational activities was associated with reduced depression symptoms, we cannot entirely discount the possibility that this association might be influenced by depression’s effect on social participation. For example, study have revealed that the higher the degree of depression, the lower the ability to participate in physical and social leisure activities [23]. Conversely, active social participation is also a therapeutic strategy for depression, and effective treatment may encourage greater involvement in social activities. Last, similar to other longitudinal studies, the CHARLS experienced attrition due to mortality and nonresponse, which could introduce sample selection bias and affect the study’s internal validity.

## Conclusions

Drawing on data from the longitudinal cohort of the CHARLS, it was observed that a higher level of social engagement pattern was correlated with excess risk of new-onset depression or a worsening depression trajectory. The consistent observation of the protective effects associated with these patterns of activity underscores the potential efficacy of these identified forms of societal participation as intervention strategies. These strategies hold promise for the primary prevention of depression within the general population, highlighting the importance of social engagement in mitigating the risk of depression and suggesting a viable pathway for public health initiatives aimed at depression prevention.

## Electronic supplementary material

Below is the link to the electronic supplementary material.

Supplementary Material 1

## Data Availability

The datasets analysed during the current study are available from the CHARLS repository [http://charls.pku.edu.cn].

## Abbreviations

ABC: Act-Belong-Commit
BMI: Body Mass Index
CES-D 10: Center for Epidemiologic Studies Depression Scale 10-item
CHARLS: China Health and Retirement Longitudinal Study
CI: Confidence Intervals
DAG: Directed Acyclic Graphs
MCA: Multiple Correspondence Analysis
IMSEC: Integrated Medical Services and Elderly Care
OR: Odds Ratios

## References

[1] GBD 2019 Diseases and Injuries Collaborators. Global burden of 369 diseases and injuries in 204 countries and territories, 1990–2019: a systematic analysis for the global burden of disease study 2019. Lancet. 2020;396:1204–22. 33069326 10.1016/S0140-6736(20)30925-9 PMC7567026

[2] ER W, RE M, Druss BG. Mortality in mental disorders and global disease burden implications: A systematic review and meta-analysis. JAMA Psychiatry. 2015;72:334–41. 25671328 10.1001/jamapsychiatry.2014.2502 PMC4461039

[3] Nicholson A, Kuper H, Hemingway H. Depression as an aetiologic and prognostic factor in coronary heart disease: a meta-analysis of 6362 events among 146 538 participants in 54 observational studies. Eur Heart J. 2006;27:2763–74. 10.1093/eurheartj/ehl338. 17082208 10.1093/eurheartj/ehl338

[4] Hasler G, Pine DS, Kleinbaum DG, Gamma A, Luckenbaugh D, Ajdacic V, et al. Depressive symptoms during childhood and adult obesity: the Zurich cohort study. Mol Psychiatry. 2005;10:842–50. 10.1038/sj.mp.4001671. 15838533 10.1038/sj.mp.4001671

[5] Patel V, Chisholm D, Parikh R, Charlson FJ, Degenhardt L, Dua T, et al. Addressing the burden of mental, neurological, and substance use disorders: key messages from disease control priorities, 3rd edition. Lancet (London England). 2016;387:1672–85. 10.1016/S0140-6736(15)00390-6. 10.1016/S0140-6736(15)00390-6 26454360

[6] Milner AJ, Carter G, Pirkis J, Robinson J, Spittal MJ. Letters, green cards, telephone calls and postcards: systematic and meta-analytic review of brief contact interventions for reducing self-harm, suicide attempts and suicide. Br J Psychiatry. 2015;206:184–90. 10.1192/bjp.bp.114.147819. 25733570 10.1192/bjp.bp.114.147819

[7] Lu J, Xu X, Huang Y, Li T, Ma C, Xu G, et al. Prevalence of depressive disorders and treatment in China: a cross-sectional epidemiological study. Lancet Psychiatry. 2021;8:981–90. 10.1016/S2215-0366(21)00251-0. 34559991 10.1016/S2215-0366(21)00251-0

[8] Hao G, Bishwajit G, Tang S, Nie C, Ji L, Huang R. Social participation and perceived depression among elderly population in South Africa. Clin Interv Aging. 2017;12:971–6. 10.2147/CIA.S137993. 28694690 10.2147/CIA.S137993 PMC5491569

[9] Santini ZI, Jose PE, York Cornwell E, Koyanagi A, Nielsen L, Hinrichsen C, et al. Social disconnectedness, perceived isolation, and symptoms of depression and anxiety among older Americans (NSHAP): a longitudinal mediation analysis. Lancet Public Heal. 2020;5:e62–70. 10.1016/S2468-2667(19)30230-0 31910981

[10] Chiao C, Weng LJ, Botticello AL. Social participation reduces depressive symptoms among older adults: an 18-year longitudinal analysis in Taiwan. BMC Public Health. 2011;11:292. 21569285 10.1186/1471-2458-11-292 PMC3103460

[11] Cheng ST, Chan ACM, Yu ECS. An exploratory study of the effect of Mahjong on the cognitive functioning of persons with dementia. Int J Geriatr Psychiatry. 2006;21:611–7. 16779765 10.1002/gps.1531

[12] Bourassa KJ, Memel M, Woolverton C, Sbarra DA. Social participation predicts cognitive functioning in aging adults over time: comparisons with physical health, depression, and physical activity. Aging Ment Heal. 2017;21:133–46. 10.1080/13607863.2015.1081152 26327492

[13] Wang R, Chen Z, Zhou Y, Shen L, Zhang Z, Wu X. Melancholy or Mahjong?? Diversity, frequency, type, and rural-urban divide of social participation and depression in middle- and old-aged Chinese: A fixed-effects analysis. Soc Sci Med. 2019;238(August):112518. 31473574 10.1016/j.socscimed.2019.112518

[14] Zhao Y, Hu Y, Smith JP, Strauss J, Yang G. Cohort profile: the China health and retirement longitudinal study (CHARLS). Int J Epidemiol. 2014;43:61–8. 23243115 10.1093/ije/dys203 PMC3937970

[15] Cotten SR, Ford G, Ford S, Hale TM. Internet use and depression among retired older adults in the united States: A longitudinal analysis. Journals Gerontol - Ser B Psychol Sci Soc Sci. 2014;69:763–71. 10.1093/geronb/gbu018 24671896

[16] Fang Y-W, Liu C-Y. Determining risk factors associated with depression and anxiety in young lung cancer patients: A novel optimization algorithm. Med (Kaunas). 2021;57. 10.3390/medicina57040340. 10.3390/medicina57040340 PMC8065798 33916080

[17] Tenenhaus MYF. An analysis and synthesis of multiple correspondence analysis, optimal scaling, dual scaling, homogeneity analysis and other methods for quantifying categorical multivariate data. Psychometrika. 1985;50:91–119.

[18] Boey KW. Cross-validation of a short form of the CES-D in Chinese elderly. Int J Geriatr Psychiatry. 1999;14:608–17. 10489651 10.1002/(sici)1099-1166(199908)14:8<608::aid-gps991>3.0.co;2-z

[19] Han T, Han M, Moreira P, Song H, Li P, Zhang Z. Association between specific social activities and depressive symptoms among older adults: A study of urban-rural differences in China. Front Public Heal. 2023;11:1099260. 10.3389/fpubh.2023.1099260. 10.3389/fpubh.2023.1099260 PMC10102908 37064675

[20] Fu H, Si L, Guo R. What is the optimal cut-off point of the 10-item center for epidemiologic studies depression scale for screening depression among Chinese individuals aged 45 and over? An exploration using latent profile analysis. Front Psychiatry. 2022;13 March:1–10. 10.3389/fpsyt.2022.820777 PMC8963942 35360127

[21] Zhu S, Kong X, Han F, Tian H, Sun S, Sun Y, et al. Association between social isolation and depression: evidence from longitudinal and Mendelian randomization analyses. J Affect Disord. 2024;350:182–7. 38220103 10.1016/j.jad.2024.01.106

[22] Perlis RH, Green J, Simonson M, Ognyanova K, Santillana M, Lin J, et al. Association between social media use and self-reported symptoms of depression in US adults. JAMA Netw Open. 2021;4:e2136113. 10.1001/jamanetworkopen.2021.36113. 34812844 10.1001/jamanetworkopen.2021.36113 PMC8611479

[23] Gao Y, Jia Z, Zhao L, Han S. The effect of activity participation in middle-aged and older people on the trajectory of depression in later life: National cohort study. JMIR Public Heal Surveill. 2023;9:e44682. 10.2196/44682. 10.2196/44682 PMC10131905 36951932

[24] Jonsdottir IH, Rödjer L, Hadzibajramovic E, Börjesson M, Ahlborg G. A prospective study of leisure-time physical activity and mental health in Swedish health care workers and social insurance officers. Prev Med (Baltim). 2010;51:373–7. 10.1016/j.ypmed.2010.07.019. 10.1016/j.ypmed.2010.07.019 20691721

[25] Qin S, Zhou M, Cheng Y, Zhao J, Ding Y. Choice preference of middle-aged and Ederly people on integrated medical services and elderly care model: A cross-sectional study. Inq (United States). 2024;61. 10.1177/00469580231224345 PMC10823860 38281995

[26] Sudo K, Kobayashi J, Noda S, Fukuda Y, Takahashi K. Japan’s healthcare policy for the elderly through the concepts of self-help (Ji-jo), mutual aid (Go-jo), social solidarity care (Kyo-jo), and governmental care (Ko-jo). Biosci Trends. 2018;12:7–11. 29479017 10.5582/bst.2017.01271

[27] Donovan RJ, Anwar-McHenry J. Act-Belong-Commit: lifestyle medicine for keeping mentally healthy. Am J Lifestyle Med. 2016;10:193–9. 30202274 10.1177/1559827614536846 PMC6124955

[28] Chen D, Yang H, Yang L, Tang Y, Zeng H, He J, et al. Preoperative psychological symptoms and chronic postsurgical pain: analysis of the prospective China surgery and anaesthesia cohort study. Br J Anaesth. 2023;132:359–71. 37953200 10.1016/j.bja.2023.10.015

[29] Croezen S, Avendano M, Burdorf A, Van Lenthe FJ. Social participation and depression in old age: A fixed-effects analysis in 10 European countries. Am J Epidemiol. 2015;182:168–76. 26025236 10.1093/aje/kwv015 PMC4493978

