# Supplementary material for: Association of social participation and patterns with depression: analysis of data from the China health and retirement longitudinal study
Source: BMC Psychiatry. 2025 Apr 4;25:335. doi: 10.1186/s12888-025-06692-9 (PMC11971768; doi:10.1186/s12888-025-06692-9)
Supplement: Supplementary file 1 — Supplementary Material 1 [file 12888_2025_6692_MOESM1_ESM.docx]

**On-line Supplementary Material**

**Content:**

[Methods 1. Details of outcome and exposure in CHARLS. 2](#_Toc156145175)

[Methods 2. Details of covariates in CHARLS 7](#_Toc156145176)

[Table S1. Characteristics of participants at baseline and stratified by the presence of new onset depression. 12](#_Toc156145177)

[Table S2. Characteristics of participants at baseline and stratified by the pattern of society participation. 14](#_Toc156145178)

[Table S3. Statistical output from latent class analysis. 16](#_Toc156145179)

[Table S4. Characteristics of participants at baseline and stratified by the trajectory of depression. 17](#_Toc156145180)

[Table S5. Risk of new onset depression among individuals with different social participation and major pattern (identified by k-means) compared with that of individuals without such conditions. 19](#_Toc156145181)

[Table S6. Risk of different depression trajectories among individuals with different social participation and major pattern (identified by k-means) compared with that of individuals without such conditions. 23](#_Toc156145182)

[Table S7. Risk of new onset depression among individuals with different social participation and major pattern (identified by k-means) compared with that of individuals without such conditions by excluding patients missing baseline depression status. 32](#_Toc156145183)

[Table S8. Risk of different depression trajectories among individuals with different social participation and major pattern (identified by k-means) compared with that of individuals without such conditions by excluding patients missing baseline depression status. 36](#_Toc156145184)

[Table S9. During the follow-up period, information on receiving treatment for depression in all new onset depression populations, and stratify by the trajectory of depression. 45](#_Toc156145185)

[Figure S1. Flowchart of participant selection. 46](#_Toc156145186)

[Figure S2. Visualization of intersecting sets of patients with preoperative psychological symptoms. 47](#_Toc156145187)

[Figure S3 The elbow plot is made by calculating the within-cluster sum of squares with k ranging from 1 to 10. 48](#_Toc156145188)

[Figure S4. The Directed Acyclic Graph of the relationship between social participation, involved covariates, and depressive statuses among participants. 49](#_Toc156145189)

[Figure S5. Risk of new onset depression and different depression trajectories among individuals with different social participation compared with that of individuals without such conditions by excluding patients missing baseline depression status. 50](#_Toc156145190)

[Figure S6. Risk of new onset depression and different depression trajectories among individuals with different social participation pattern (identified by k-means) compared with that of individuals without such conditions by excluding patients missing baseline depression status. 51](#_Toc156145191)

## Methods 1. Details of outcome and exposure in CHARLS.

| **Covariates** | **Item numbers in the questionnaire** | **Questionnaire items and definitions** | **Responses transformation** |
| --- | --- | --- | --- |
| Social leisure activity participation | | | |
| Social leisure activity participation | DA056 (Wave 1) | Have you done any of these activities in the last month? (Code all that apply) Interacted with friends? (1,0) | **1** = Yes  **0** = No |
|  |  | Have you done any of these activities in the last month? (Code all that apply) Played Ma-jong, played chess, played cards, or went to community club? (1,0) | **1** = Yes  **0** = No |
|  |  | Have you done any of these activities in the last month? (Code all that apply) Provided help to family, friends, or neighbors who do not live with you and who did not pay you for the help? (1,0) | **1** = Yes  **0** = No |
|  |  | Have you done any of these activities in the last month? (Code all that apply) Went to a sport, social, or other kind of club? (1,0) | **1** = Yes  **0** = No |
|  |  | Have you done any of these activities in the last month? Took part in a community-related organization? (1,0) | **1** = Yes  **0** = No |
|  |  | Have you done any of these activities in the last month? (Code all that apply) Done voluntary or charity work? (1,0) | **1** = Yes  **0** = No |
|  |  | Have you done any of these activities in the last month? (Code all that apply) Cared for a sick or disabled adult who does not live with you and who did not pay you for the help? (1,0) | **1** = Yes  **0** = No |
|  |  | Have you done any of these activities in the last month? (Code all that apply) Attended an educational or training course? (1,0) | **1** = Yes  **0** = No |
|  |  | Have you done any of these activities in the last month? (Code all that apply) Stock investment? (1,0) | **1** = Yes  **0** = No |
|  |  | Have you done any of these activities in the last month? (Code all that apply) Used the Internet? (1,0) | **1** = Yes  **0** = No |
|  |  | Have you done any of these activities in the last month? (Code all that apply) Other? (1,0) | **1** = Yes  **0** = No |
|  | DA057 (Wave 1) | How often in the last month [did/have][you] [do voluntary or charity work/cared for a sick or disabled adult/provided help to family, friends or neighbors/attended an educational or training course/ Interacted with friends /go to a sport,social or other kind of club/taken part in a community-related organization]? Almost daily, almost every week, or not regularly? (1,2,3) | **0 =** This activity is not carried out  **1** = Almost daily  **2** = Almost every week  **3** = Not regularly |
| **CES-D scores** | | | |
| CES-D | DC009 (Wave 1 - 5) | I was bothered by things that don't usually bother me. (0,1,2,3) | **0** = Rarely or none of the time (<1 day)  **1** = Some or a little of the time (1-2 days)  **2** = Occasionally or a moderate amount of the time (3-4 days)  **3** = Most or all of the time (5-7 days) |
|  | DC010 (Wave 1 - 5) | I had trouble keeping my mind on what I was doing. (0,1,2,3) | **0** = Rarely or none of the time (<1 day)  **1** = Some or a little of the time (1-2 days)  **2** = Occasionally or a moderate amount of the time (3-4 days)  **3** = Most or all of the time (5-7 days) |
|  | DC011 (Wave 1 - 5) | I felt depressed. (0,1,2,3) | **0** = Rarely or none of the time (<1 day)  **1** = Some or a little of the time (1-2 days)  **2** = Occasionally or a moderate amount of the time (3-4 days)  **3** = Most or all of the time (5-7 days) |
|  | DC012 (Wave 1 - 5) | I felt everything I did was an effort. (0,1,2,3) | **0** = Rarely or none of the time (<1 day)  **1** = Some or a little of the time (1-2 days)  **2** = Occasionally or a moderate amount of the time (3-4 days)  **3** = Most or all of the time (5-7 days) |
|  | DC013 (Wave 1 - 5) | I felt hopeful about the future. (0,1,2,3) | **0** = Most or all of the time (5-7 days)  **1** = Occasionally or a moderate amount of the time (3-4 days)  **2** = Some or a little of the time (1-2 days)  **3** = Rarely or none of the time (<1 day) |
|  | DC014 (Wave 1 - 5) | I felt fearful. (0,1,2,3) | **0** = Rarely or none of the time (<1 day)  **1** = Some or a little of the time (1-2 days)  **2** = Occasionally or a moderate amount of the time (3-4 days)  **3** = Most or all of the time (5-7 days) |
|  | DC015 (Wave 1 - 5) | My sleep was restless. (0,1,2,3) | **0** = Rarely or none of the time (<1 day)  **1** = Some or a little of the time (1-2 days)  **2** = Occasionally or a moderate amount of the time (3-4 days)  **3** = Most or all of the time (5-7 days) |
|  | DC016 (Wave 1 - 5) | I was happy. (0,1,2,3) | **0** = Most or all of the time (5-7 days)  **1** = Occasionally or a moderate amount of the time (3-4 days)  **2** = Some or a little of the time (1-2 days)  **3** = Rarely or none of the time (<1 day) |
|  | DC017 (Wave 1 - 5) | I felt lonely. (0,1,2,3) | **0** = Rarely or none of the time (<1 day)  **1** = Some or a little of the time (1-2 days)  **2** = Occasionally or a moderate amount of the time (3-4 days)  **3** = Most or all of the time (5-7 days) |
|  | DC018 (Wave 1 - 5) | I could not get "going." (0,1,2,3) | **0** = Rarely or none of the time (<1 day)  **1** = Some or a little of the time (1-2 days)  **2** = Occasionally or a moderate amount of the time (3-4 days)  **3** = Most or all of the time (5-7 days) |
| **Treatment of illness** | | | |
| Treatment | DA020 (Wave 1 - 4) | Are you now taking any of the following treatments for your emotional, nervous, or psychiatric problems? (Check all that apply) Receiving psychiatric or psychological treatment, taking anti- depressants, taking tranquilizers or sleeping pills? Receiving psychiatric or psychological treatment? | **1** = Yes  **0** = No |
|  |  | Are you now taking any of the following treatments for your emotional, nervous, or psychiatric problems? (Check all that apply) Receiving psychiatric or psychological treatment, taking anti- depressants ,taking tranquilizers or sleeping pills? Taking anti-depressants? | **1** = Yes  **0** = No |
|  |  | Are you now taking any of the following treatments for your emotional, nervous, or psychiatric problems? (Check all that apply) Receiving psychiatric or psychological treatment, taking anti- depressants, taking tranquilizers or sleeping pills? Taking tranquilizers or sleeping pills? | **1** = Yes  **0** = No |
|  |  | Are you now taking any of the following treatments for your emotional, nervous, or psychiatric problems? (Check all that apply) Receiving psychiatric or psychological treatment, taking anti- depressants, taking tranquilizers or sleeping pills? None of the above? | **1** = Yes  **0** = No |

## Methods 2. Details of covariates in CHARLS

| **Covariates** | **Item numbers in the questionnaire** | **Questionnaire items and definitions** | **Responses transformation** |
| --- | --- | --- | --- |
| Demographic characteristic | | | |
| Age | BA002 (Wave 1 - 2) | When were you born? | Continuous variable;  Categorical variable: 50; 51-59; 60 |
|  | BA004 (Wave 1) | What is your age? |  |
|  | BA002 (Wave 3 - 4) | What’s your actual date of birth? |  |
|  | BA004_W3 (Wave 3 - 4) | What’s your date of birth on ID card or Household register? |  |
| Gender | CV005 (Wave 1) | Record gender of main respondent. (Male, Female) | **Male**=Male  **Female**=Female |
|  | BA000_W2_3 (Wave 2 - 4) | Interviewer record R’s gender. (Male, Female) |  |
| Body Mass Index ^a^ | PL002 (Wave 1) | Record weight measurement. | Continuous variable |
|  | QL002 (Wave 1) | Record height measurement. |  |
| Marital status | BE001 (Wave 1) | What is your marital status? (Single, Married or cohabiting, Divorced or widowed) | **Single** = Never married  **Married or cohabiting** = Married with spouse present; Married but not living with spouse temporarily for reasons such as work  **Divorced or widowed** = Separated; Divorced; Widowed |
| Educational level | BD001 (Wave 1) | What is the highest level of education you have attained? (Lower secondary, Tertiary, Upper secondary and vocational training) | **Lower secondary** = No formal education; Did not finish primary school but capable of reading and/or writing; Sishu/home school; Elementary school; Middle school  **Tertiary** = High school; Vocational school  **Upper secondary and vocational training** = Two-/Three-Year College/Associate degree; Four-Year College/Bachelor’s degree; Master’s degree; Doctoral degree/Ph.D. |
| Residence | A001 (Wave 1) | Take down the type of this neighbourhood. (Rural, Urban) | **Rural** = Rural Village  **Urban** = Urban Community |
| Living type | | | |
| Smoking status | DA061 (Wave 1) | Do you still have the habit or have you totally quit? (Ever, Current) | **Ever** = Quit  **Current** = Still have |
|  | DA059 (Wave 1- 4) | Have you ever chewed tobacco, smoked a pipe, smoked self-rolled cigarettes, or smoked cigarettes/cigars? (Never) | **Never** = No |
| Drinking status | DA067 (Wave 1) | Did you drink any alcoholic beverages, such as beer, wine, or liquor in the past year? How often? (Current, Never) | **Current** = Drink more than once a month；Drink but less than once a month  **Never** = None of these |
|  | DA069 (Wave 1- 4) | Did you ever drink alcoholic beverages in the past? How often? (Never) | **Never** = I never had a drink |
| Chronic disease | | | |
| Hypertension | DA007 (Wave 1) | Have you been diagnosed with hypertension by a doctor? (Yes, No) | **Yes** = Yes  **No** = No |
| Diabetes |  | Have you been diagnosed with diabetes or high blood sugar by a doctor? (Yes, No) | **Yes** = Yes  **No** = No |
| Dyslipidemia |  | Have you been diagnosed with dyslipidemia by a doctor? (Yes, No) | **Yes** = Yes  **No** = No |
| Heart disease |  | Have you been diagnosed with heart attack, coronary heart disease, angina, congestive heart failure, or other heart problems by a doctor? (Yes, No) | **Yes** = Yes  **No** = No |
| Respiratory illness |  | Have you been diagnosed with chronic lung diseases / asthma by a doctor? (Yes, No) | **Yes** = Yes  **No** = No |
| Liver disease |  | Have you been diagnosed with liver disease by a doctor? (Yes, No) | **Yes** = Yes  **No** = No |
| Kidney disease |  | Have you been diagnosed with kidney disease (except for tumor or cancer) by a doctor? (Yes, No) | **Yes** = Yes  **No** = No |
| Digestive disease |  | Have you been diagnosed with stomach or other digestive disease (except for tumor or cancer) by a doctor? (Yes, No) | **Yes** = Yes  **No** = No |
| Arthritis or rheumatism |  | Have you been diagnosed with arthritis or rheumatism by a doctor? (Yes, No) | **Yes** = Yes  **No** = No |
| Cancer |  | Have you been diagnosed with cancer or malignant tumor (excluding minor skin cancers) by a doctor? (Yes, No) | **Yes** = Yes  **No** = No |
| Basic activities of daily living | | | |
| Continence | DB003 (Wave 1) | Do you have difficulty walking 1 km? (1,0) | **1** = No, I don’t have any difficulty; I have difficulty but can still do it.  **0** = Yes, I have difficulty and need help; I can not do it. |
| Dressing | DB010 (Wave 1) | Do you have any difficulty with dressing? (1,0) | **1** = No, I don’t have any difficulty; I have difficulty but can still do it.  **0** = Yes, I have difficulty and need help; I can not do it. |
| Bathing | DB011 (Wave 1) | Do you have any difficulty with bathing or showering? (1,0) | **1** = No, I don’t have any difficulty; I have difficulty but can still do it.  **0** = Yes, I have difficulty and need help; I can not do it. |
| Feeding | DB012 (Wave 1) | Do you have any difficulty with eating? (1,0) | **1** = No, I don’t have any difficulty; I have difficulty but can still do it.  **0** = Yes, I have difficulty and need help; I can not do it. |
| Transferring | DB013 (Wave 1) | Do you have any difficulty with getting into or out of bed? (1,0) | **1** = No, I don’t have any difficulty; I have difficulty but can still do it.  **0** = Yes, I have difficulty and need help; I can not do it. |
| Going to toilet | DB014 (Wave 1) | Do you have any difficulties with using the toilet? (1,0) | **1** = No, I don’t have any difficulty; I have difficulty but can still do it.  **0** = Yes, I have difficulty and need help; I can not do it. |
| Basic activities of daily living difficulty | DB003, DB010, DB011, DB012, DB03, DB014 (Wave 1) | Do you have difficulty walking 1 km?; Do you have any difficulty with dressing?; Do you have any difficulty with bathing or showering?; Do you have any difficulty with bathing or showering?; Do you have any difficulty with eating?; Do you have any difficulties with using the toilet? (1,0) | **1** = any of basic activities of daily living scores = 0  **0** = sum of basic activities of daily living scores = 6 |
| Instrumental activities of daily living | | | |
| Housekeeping | DB016 (Wave 1) | Because of health and memory problems, do you have any difficulties with doing household chores? (1,0) | **1** = No, I don’t have any difficulty; I have difficulty but can still do it.  **0** = Yes, I have difficulty and need help; I can not do it. |
| Preparing hot meals | DB017 (Wave 1) | Because of health and memory problems, do you have any difficulties with preparing hot meals? (1,0) | **1** = No, I don’t have any difficulty; I have difficulty but can still do it.  **0** = Yes, I have difficulty and need help; I can not do it. |
| Shopping | DB018 (Wave 1) | Because of health and memory problems, do you have any difficulties with shopping for groceries? By shopping, we mean deciding what to buy and paying for it. (1,0) | **1** = No, I don’t have any difficulty; I have difficulty but can still do it.  **0** = Yes, I have difficulty and need help; I can not do it. |
| Managing money | DB019 (Wave 1) | Because of health and memory problems, do you have any difficulties with managing your money, such as paying your bills, keeping track of expenses, or managing assets? (1,0) | **1** = No, I don’t have any difficulty; I have difficulty but can still do it.  **0** = Yes, I have difficulty and need help; I can not do it. |
| Taking medications | DB020 (Wave 1) | Because of health and memory problems, do you have any difficulties with taking medications? By taking medications, we mean taking the right portion of medication right on time? (1,0) | **1** = No, I don’t have any difficulty; I have difficulty but can still do it.  **0** = Yes, I have difficulty and need help; I can not do it. |
| Instrumental activities of daily living difficulty | DB016; DB017; DB018; DB019; DB020 (Wave 1) | Because of health and memory problems, do you have any difficulties with doing household chores?; Because of health and memory problems, do you have any difficulties with preparing hot meals?; Because of health and memory problems, do you have any difficulties with shopping for groceries? By shopping, we mean deciding what to buy and paying for it.; Because of health and memory problems, do you have any difficulties with managing your money, such as paying your bills, keeping track of expenses, or managing assets?; Because of health and memory problems, do you have any difficulties with taking medications? By taking medications, we mean taking the right portion of medication right on time? (1,0) | **1** = any of instrumental activities of daily living scores = 0  **0** = sum of instrumental activities of daily living scores = 5 |

1. Body Mass Index = weight (kg) / height(m)^2^

## Table S1. Characteristics of participants at baseline and stratified by the presence of new onset depression.

|  | **Total** | **New onset depression** | **Without new onset depression** | ***P*** |
| --- | --- | --- | --- | --- |
| **No. of participants** | **9415** | **4348** | **5067** |  |
| Age, yrs, mean (SD) | 57.87 (9.27) | 57.62 (9.06) | 58.08 (9.44) | 0.016 |
| Male, No. (%) | 4831 (51.3) | 1918 (44.1) | 2913 (57.5) | <0.001 |
| Body mass index, kg/m^2^, mean (SD) | 24.09 (12.81) | 24.17 (15.27) | 24.02 (10.14) | 0.599 |
| Marital status, No. (%) |  |  |  | 0.023 |
| Divorced or widowed | 797 (8.5) | 394 (9.1) | 403 (8.0) |  |
| Married or cohabiting | 8562 (90.9) | 3921 (90.2) | 4641 (91.6) |  |
| Single | 56 (0.6) | 33 (0.8) | 23 (0.5) |  |
| Education, No. (%) |  |  |  | <0.001 |
| Lower secondary | 7978 (84.7) | 3909 (89.9) | 4069 (80.3) |  |
| Upper secondary and vocational training | 1162 (12.3) | 383 (8.8) | 779 (15.4) |  |
| Tertiary | 275 (2.9) | 56 (1.3) | 219 (4.3) |  |
| Current employment status, No. (%) |  |  |  | <0.001 |
| Retire | 1342 (14.3) | 440 (10.1) | 902 (17.8) |  |
| Farmer | 438 (4.7) | 214 (4.9) | 224 (4.4) |  |
| Individual firm | 1044 (11.1) | 390 (9.0) | 654 (12.9) |  |
| Government or institutions | 361 (3.8) | 112 (2.6) | 249 (4.9) |  |
| Unknown | 6230 (66.2) | 3192 (73.4) | 3038 (60.0) |  |
| Residence, No. (%) |  |  |  | <0.001 |
| Rural | 4376 (46.5) | 2238 (51.5) | 2138 (42.2) |  |
| Urban | 3459 (36.7) | 1306 (30.0) | 2153 (42.5) |  |
| Unknown | 1580 (16.8) | 804 (18.5) | 776 (15.3) |  |
| Smoking status, No. (%) |  |  |  | <0.001 |
| Current | 3010 (32.0) | 1268 (29.2) | 1742 (34.4) |  |
| Ever | 805 (8.6) | 307 (7.1) | 498 (9.8) |  |
| Never | 5600 (59.5) | 2773 (63.8) | 2827 (55.8) |  |
| Non-alcohol consumption, No. (%) | 6036 (64.1) | 2971 (68.3) | 3065 (60.5) | <0.001 |
| Hypertension, No. (%) | 2070 (22.0) | 973 (22.4) | 1097 (21.6) | 0.409 |
| Diabetes mellitus, No. (%) | 462 (4.9) | 226 (5.2) | 236 (4.7) | 0.245 |
| Dyslipidemia, No. (%) | 817 (8.7) | 349 (8.0) | 468 (9.2) | 0.041 |
| Heart disease, No. (%) | 864 (9.2) | 412 (9.5) | 452 (8.9) | 0.371 |
| Respiratory disease, No. (%) | 815 (8.7) | 405 (9.3) | 410 (8.1) | 0.039 |
| Liver disease, No. (%) | 301 (3.2) | 133 (3.1) | 168 (3.3) | 0.518 |
| Kidney disease, No. (%) | 456 (4.8) | 251 (5.8) | 205 (4.0) | <0.001 |
| Digestive disease, No. (%) | 1753 (18.6) | 949 (21.8) | 804 (15.9) | <0.001 |
| Arthritis or rheumatism, No. (%) | 2587 (27.5) | 1383 (31.8) | 1204 (23.8) | <0.001 |
| Cancer, No. (%) | 76 (0.8) | 40 (0.9) | 36 (0.7) | 0.309 |
| Difﬁculty in BADL, No. (%) | 457 (4.9) | 252 (5.8) | 205 (4.0) | <0.001 |
| Difﬁculty in IADL, No. (%) | 629 (6.7) | 360 (8.3) | 269 (5.3) | <0.001 |
| Baseline CES-D 10, mean (SD) | 4.34 (2.78) | 5.08 (2.69) | 3.75 (2.71) | <0.001 |

The values are reported as the mean (standard deviation) for continuous variables and n (%) for categorical variables.

Abbreviation: BADL: basic activities of daily living; CES-D 10: Epidemiologic Studies Depression Scale 10; IADL: instrumental activities of daily living

## Table S2. Characteristics of participants at baseline and stratified by the pattern of society participation.

|  | **Pattern 1** | **Pattern 2** | **Pattern 3** | **Pattern 4** | **Pattern 5** | **Pattern 6** | ***P*** |
| --- | --- | --- | --- | --- | --- | --- | --- |
| **No. of participants** | 4429 | 1384 | 945 | 922 | 1426 | 309 |  |
| Age, yrs, mean (SD) | 58.25 (9.14) | 58.02 (9.69) | 56.77 (8.94) | 57.07 (9.03) | 58.64 (9.53) | 53.80 (8.28) | <0.001 |
| Male, No. (%) | 2251 (50.8) | 612 (44.2) | 515 (54.5) | 517 (56.1) | 767 (53.8) | 169 (54.7) | <0.001 |
| Body mass index, kg/m^2^, mean (SD) | 23.55 (7.99) | 23.97 (3.78) | 24.42 (19.55) | 24.13 (4.54) | 25.54 (24.61) | 24.85 (4.21) | <0.001 |
| Marital status, No. (%) |  |  |  |  |  |  | 0.076 |
| Divorced or widowed | 370 (8.4) | 142 (10.3) | 71 (7.5) | 66 (7.2) | 125 (8.8) | 23 (7.4) |  |
| Married or cohabiting | 4024 (90.9) | 1236 (89.3) | 868 (91.9) | 851 (92.3) | 1298 (91.0) | 285 (92.2) |  |
| Single | 35 (0.8) | 6 (0.4) | 6 (0.6) | 5 (0.5) | 3 (0.2) | 1 (0.3) |  |
| Education, No. (%) |  |  |  |  |  |  | <0.001 |
| Lower secondary | 3955 (89.3) | 1264 (91.3) | 794 (84.0) | 795 (86.2) | 1067 (74.8) | 103 (33.3) |  |
| Upper secondary and vocational training | 410 (9.3) | 107 (7.7) | 138 (14.6) | 110 (11.9) | 279 (19.6) | 118 (38.2) |  |
| Tertiary | 64 (1.4) | 13 (0.9) | 13 (1.4) | 17 (1.8) | 80 (5.6) | 88 (28.5) |  |
| Current employment status, No. (%) |  |  |  |  |  |  | <0.001 |
| Retire | 447 (10.1) | 135 (9.8) | 121 (12.8) | 93 (10.1) | 433 (30.4) | 113 (36.6) |  |
| Farmer | 198 (4.5) | 67 (4.8) | 59 (6.2) | 54 (5.9) | 56 (3.9) | 4 (1.3) |  |
| Individual firm | 505 (11.4) | 127 (9.2) | 118 (12.5) | 101 (11.0) | 131 (9.2) | 62 (20.1) |  |
| Government or institutions | 135 (3.0) | 26 (1.9) | 33 (3.5) | 34 (3.7) | 77 (5.4) | 56 (18.1) |  |
| Unknown | 3144 (71.0) | 1029 (74.3) | 614 (65.0) | 640 (69.4) | 729 (51.1) | 74 (23.9) |  |
| Residence, No. (%) |  |  |  |  |  |  | <0.001 |
| Rural | 2158 (48.7) | 767 (55.4) | 488 (51.6) | 464 (50.3) | 468 (32.8) | 31 (10.0) |  |
| Urban | 1477 (33.3) | 412 (29.8) | 309 (32.7) | 285 (30.9) | 743 (52.1) | 233 (75.4) |  |
| Unknown | 794 (17.9) | 205 (14.8) | 148 (15.7) | 173 (18.8) | 215 (15.1) | 45 (14.6) |  |
| Smoking status, No. (%) |  |  |  |  |  |  | <0.001 |
| Current | 1373 (31.0) | 425 (30.7) | 335 (35.4) | 347 (37.6) | 444 (31.1) | 86 (27.8) |  |
| Ever | 356 (8.0) | 95 (6.9) | 98 (10.4) | 81 (8.8) | 144 (10.1) | 31 (10.0) |  |
| Never | 2700 (61.0) | 864 (62.4) | 512 (54.2) | 494 (53.6) | 838 (58.8) | 192 (62.1) |  |
| Non-alcohol consumption, No. (%) | 2960 (66.8) | 935 (67.6) | 564 (59.7) | 559 (60.6) | 858 (60.2) | 160 (51.8) | <0.001 |
| Hypertension, No. (%) | 905 (20.4) | 333 (24.1) | 176 (18.6) | 192 (20.8) | 378 (26.5) | 86 (27.8) | <0.001 |
| Diabetes mellitus, No. (%) | 190 (4.3) | 71 (5.1) | 39 (4.1) | 41 (4.4) | 100 (7.0) | 21 (6.8) | 0.001 |
| Dyslipidemia, No. (%) | 312 (7.0) | 125 (9.0) | 75 (7.9) | 75 (8.1) | 176 (12.3) | 54 (17.5) | <0.001 |
| Heart disease, No. (%) | 400 (9.0) | 117 (8.5) | 71 (7.5) | 74 (8.0) | 172 (12.1) | 30 (9.7) | 0.001 |
| Respiratory disease, No. (%) | 390 (8.8) | 106 (7.7) | 88 (9.3) | 92 (10.0) | 119 (8.3) | 20 (6.5) | 0.264 |
| Liver disease, No. (%) | 132 (3.0) | 51 (3.7) | 32 (3.4) | 21 (2.3) | 49 (3.4) | 16 (5.2) | 0.129 |
| Kidney disease, No. (%) | 208 (4.7) | 67 (4.8) | 47 (5.0) | 41 (4.4) | 73 (5.1) | 20 (6.5) | 0.766 |
| Digestive disease, No. (%) | 861 (19.4) | 236 (17.1) | 175 (18.5) | 183 (19.8) | 243 (17.0) | 55 (17.8) | 0.178 |
| Arthritis or rheumatism, No. (%) | 1264 (28.5) | 393 (28.4) | 285 (30.2) | 243 (26.4) | 350 (24.5) | 52 (16.8) | <0.001 |
| Cancer, No. (%) | 36 (0.8) | 10 (0.7) | 5 (0.5) | 7 (0.8) | 16 (1.1) | 2 (0.6) | 0.711 |
| Difﬁculty in BADL, No. (%) | 291 (6.6) | 54 (3.9) | 44 (4.7) | 23 (2.5) | 43 (3.0) | 2 (0.6) | <0.001 |
| Difﬁculty in IADL, No. (%) | 381 (8.6) | 83 (6.0) | 68 (7.2) | 42 (4.6) | 51 (3.6) | 4 (1.3) | <0.001 |
| Baseline CES-D 10, mean (SD) | 4.55 (2.79) | 4.44 (2.74) | 4.54 (2.81) | 4.14 (2.72) | 3.86 (2.76) | 3.36 (2.54) | <0.001 |

The values are reported as the mean (standard deviation) for continuous variables and n (%) for categorical variables.

Abbreviation: BADL: basic activities of daily living; CES-D 10: Epidemiologic Studies Depression Scale 10; IADL: instrumental activities of daily living.

## Table S3. Statistical output from latent class analysis.

|  | **Statistic ^a^** | | | **Class size ^b^ N, (%),**  **(Posterior probabilities in each class, %)** | | | | | |
| --- | --- | --- | --- | --- | --- | --- | --- | --- | --- |
| **Class number** | **AIC ^a^** | **BIC ^a^** | **Entropy ^a^** | **1** | **2** | **3** | **4** | **5** | **6** |
| **2** | 215577.4 | 215663.2 | 0.7458549 | 8083 | 1332 | - | - | - | - |
|  |  |  |  | (85.85236) | (14.14764) |  |  |  |  |
|  |  |  |  | (94.49) | (84.93) |  |  |  |  |
| **3** | 214765.1 | 214886.6 | 0.6517459 | 6942 | 1548 | 925 | - | - | - |
|  |  |  |  | (73.73340) | (16.44185) | (9.824748) |  |  |  |
|  |  |  |  | (87.76) | (81.00) | (75.14) |  |  |  |
| **4** | 214408.7 | 214566.1 | 0.5584416 | 2920 | 4852 | 623 | 1020 | - | - |
|  |  |  |  | (31.01434) | (51.53478) | (6.617100) | (10.833776) |  |  |
|  |  |  |  | (75.50) | (79.25) | (74.27) | (81.53) |  |  |
| **5** | 214162.0 | 214355.0 | 0.5760984 | 4577 | 2617 | 1354 | 598 | 269 | - |
|  |  |  |  | (48.61391) | (27.79607) | (14.381306) | (6.351567) | (2.857143) |  |
|  |  |  |  | (79.08) | (60.12) | (71.64) | (73.60) | (78.74) |  |
| **6** | 214381.1 | 214609.9 | 0.3979019 | 0 | 3824 | 3045 | 878 | 477 | 1191 |
|  |  |  |  |  | (40.61604) | (32.342007) | (9.325544) | (5.066383) | (12.65003) |
|  |  |  |  |  | (42.85) | (47.68) | (54.61) | (67.06) | (76.54) |

^a^ AIC and BIC are information criteria for comparing models, where lower value suggests a better fit; Entropy is a measure between 0 and 1 that measures the success of classification, where a value closer to 1 implies a better fit.

^b^ class size shows the number of samples assigned to each cluster, and a relatively large size of each cluster is preferred.

Abbreviations: AIC, Akaike information criterion; BIC, Bayesian information criteria.

## Table S4. Characteristics of participants at baseline and stratified by the trajectory of depressive trajectories.

|  | **Class 1**  **“Stable low symptoms”** | **Class 2**  **“Slight increase symptoms”** | **Class 3**  **“Gradual increase symptoms”** | **Class 4**  **“Sharp worsening and persist symptoms”** | ***P*** |
| --- | --- | --- | --- | --- | --- |
| **No. of participants** | 4852 | 2920 | 623 | 1020 |  |
| Age, yrs, mean (SD) | 57.61 (9.24) | 58.09 (9.39) | 57.10 (8.55) | 58.91 (9.41) | <0.001 |
| Male, No. (%) | 2779 (57.3) | 1441 (49.3) | 254 (40.8) | 357 (35.0) | <0.001 |
| Body mass index, kg/m^2^, mean (SD) | 24.24 (13.48) | 24.11 (14.80) | 24.13 (4.20) | 23.33 (4.05) | 0.300 |
| Marital status, No. (%) |  |  |  |  | <0.001 |
| Divorced or widowed | 355 (7.3) | 265 (9.1) | 62 (10.0) | 115 (11.3) |  |
| Married or cohabiting | 4479 (92.3) | 2634 (90.2) | 556 (89.2) | 893 (87.5) |  |
| Single | 18 (0.4) | 21 (0.7) | 5 (0.8) | 12 (1.2) |  |
| Education, No. (%) |  |  |  |  | <0.001 |
| Lower secondary | 3861 (79.6) | 2586 (88.6) | 579 (92.9) | 952 (93.3) |  |
| Upper secondary and vocational training | 779 (16.1) | 285 (9.8) | 40 (6.4) | 58 (5.7) |  |
| Tertiary | 212 (4.4) | 49 (1.7) | 4 (0.6) | 10 (1.0) |  |
| Current employment status, No. (%) |  |  |  |  | <0.001 |
| Retire | 859 (17.7) | 351 (12.0) | 50 (8.0) | 82 (8.0) |  |
| Farmer | 219 (4.5) | 138 (4.7) | 29 (4.7) | 52 (5.1) |  |
| Individual firm | 647 (13.3) | 276 (9.5) | 62 (10.0) | 59 (5.8) |  |
| Government or institutions | 243 (5.0) | 89 (3.0) | 15 (2.4) | 14 (1.4) |  |
| Unknown | 2884 (59.4) | 2066 (70.8) | 467 (75.0) | 813 (79.7) |  |
| Residence, No. (%) |  |  |  |  | <0.001 |
| Rural | 2065 (42.6) | 1446 (49.5) | 324 (52.0) | 541 (53.0) |  |
| Urban | 2053 (42.3) | 948 (32.5) | 191 (30.7) | 267 (26.2) |  |
| Unknown | 734 (15.1) | 526 (18.0) | 108 (17.3) | 212 (20.8) |  |
| Smoking status, No. (%) |  |  |  |  | <0.001 |
| Current | 1668 (34.4) | 913 (31.3) | 168 (27.0) | 261 (25.6) |  |
| Ever | 448 (9.2) | 259 (8.9) | 45 (7.2) | 53 (5.2) |  |
| Never | 2736 (56.4) | 1748 (59.9) | 410 (65.8) | 706 (69.2) |  |
| Non-alcohol consumption, No. (%) | 2949 (60.8) | 1886 (64.6) | 435 (69.8) | 766 (75.1) | <0.001 |
| Hypertension, No. (%) | 1014 (20.9) | 654 (22.4) | 137 (22.0) | 265 (26.0) | 0.004 |
| Diabetes mellitus, No. (%) | 219 (4.5) | 148 (5.1) | 34 (5.5) | 61 (6.0) | 0.196 |
| Dyslipidemia, No. (%) | 446 (9.2) | 231 (7.9) | 44 (7.1) | 96 (9.4) | 0.088 |
| Heart disease, No. (%) | 416 (8.6) | 274 (9.4) | 53 (8.5) | 121 (11.9) | 0.010 |
| Respiratory disease, No. (%) | 358 (7.4) | 291 (10.0) | 46 (7.4) | 120 (11.8) | <0.001 |
| Liver disease, No. (%) | 157 (3.2) | 94 (3.2) | 16 (2.6) | 34 (3.3) | 0.829 |
| Kidney disease, No. (%) | 188 (3.9) | 159 (5.4) | 33 (5.3) | 76 (7.5) | <0.001 |
| Digestive disease, No. (%) | 761 (15.7) | 592 (20.3) | 139 (22.3) | 261 (25.6) | <0.001 |
| Arthritis or rheumatism, No. (%) | 1091 (22.5) | 910 (31.2) | 197 (31.6) | 389 (38.1) | <0.001 |
| Cancer, No. (%) | 34 (0.7) | 27 (0.9) | 6 (1.0) | 9 (0.9) | 0.694 |
| Difﬁculty in BADL, No. (%) | 175 (3.6) | 142 (4.9) | 31 (5.0) | 109 (10.7) | <0.001 |
| Difﬁculty in IADL, No. (%) | 238 (4.9) | 199 (6.8) | 53 (8.5) | 139 (13.6) | <0.001 |
| Baseline CES-D 10, mean (SD) | 3.43 (2.60) | 5.33 (2.57) | 5.00 (2.73) | 5.76 (2.61) | <0.001 |

The values are reported as the mean (standard deviation) for continuous variables and n (%) for categorical variables.

Abbreviation: BADL: basic activities of daily living; CES-D 10: Epidemiologic Studies Depression Scale 10; IADL: instrumental activities of daily living.

## Table S5. Risk of new onset depression among individuals with different social participation and major pattern (identified by k-means) compared with that of individuals without such conditions.

| **Social activities** | **No. of cases/ total (%)** | **Crude OR (95% CI)** | **Adjusted OR (95% CI)** | **p** |
| --- | --- | --- | --- | --- |
| ***Individual activities*** |  |  |  |  |
| Interact with friends |  |  |  |  |
| No | 2807/5988 (46.9) | ref | ref |  |
| Yes | 1541/3427 (45.0) | 0.93 (0.85, 1.01) | 0.90 (0.83, 0.99) | <0.001 |
| Frequency of interact with friends |  |  |  |  |
| None | 2807/5988 (46.9) | ref | ref |  |
| Not regularly | 754/1692 (44.6) | 0.91 (0.82, 1.02) | 0.85 (0.76, 0.95) | 0.005 |
| 1/week | 353/773 (45.7) | 0.95 (0.82, 1.11) | 1.00 (0.86, 0.95) | 0.967 |
| Daily | 434/962 (45.1) | 0.93 (0.81, 1.07) | 0.93 (0.80, 1.07) | 0.296 |
| Recreational (mah-jong, cards, chess, etc.) |  |  |  |  |
| No | 3554/7383 (48.1) | ref | ref |  |
| Yes | 794/2032 (39.1) | 0.69 (0.63, 0.76) | 0.79 (0.71, 0.87) | <0.001 |
| Frequency of recreational |  |  |  |  |
| None | 3554/7383 (48.1) | ref | ref |  |
| Not regularly | 182/538 (33.8) | 0.55 (0.46, 0.66) | 0.65 (0.54, 0.79) | <0.001 |
| 1/week | 258/695 (37.1) | 0.64 (0.54, 0.75) | 0.73 (0.63, 0.86) | <0.001 |
| Daily | 354/799 (44.3) | 0.86 (0.74, 0.99) | 0.94 (0.81, 1.10) | 0.444 |
| Provide help to others |  |  |  |  |
| No | 4059/8729 (46.5) | ref | ref |  |
| Yes | 289/686 (42.1) | 0.84 (0.72, 0.98) | 0.93 (0.78, 1.09) | 0.357 |
| Frequency of provide help to others |  |  |  |  |
| None | 4059/8729 (46.5) | ref | ref |  |
| Not regularly | 29/69 (42.0) | 0.83 (0.51, 1.34) | 0.89 (0.54, 1.47) | 0.657 |
| 1/week | 40/122 (32.8) | 0.56 (0.38, 0.82) | 0.70 (0.47, 1.03) | 0.078 |
| Daily | 220/495 (44.4) | 0.92 (0.77, 1.10) | 0.99 (0.82, 1.20) | 0.923 |
| Attend team sports |  |  |  |  |
| No | 4129/8676 (47.6) | ref | ref |  |
| Yes | 219/739 (29.6) | 0.46 (0.39, 0.55) | 0.70 (0.58, 0.83) | <0.001 |
| Frequency of attend team sports |  |  |  |  |
| None | 4129/8676 (47.6) | ref | ref |  |
| Not regularly | 144/488 (29.5) | 0.46 (0.38, 0.56) | 0.70 (0.57, 0.87) | 0.001 |
| 1/week | 43/148 (29.1) | 0.45 (0.31, 0.64) | 0.68 (0.47, 0.98) | 0.043 |
| Daily | 32/103 (31.1) | 0.50 (0.32, 0.75) | 0.69 (0.44, 1.06) | 0.101 |
| Community organization |  |  |  |  |
| No | 4294/9243 (46.5) | ref | ref |  |
| Yes | 54/172 (31.4) | 0.53 (0.38, 0.73) | 0.78 (0.55, 1.09) | 0.153 |
| Frequency of community organization |  |  |  |  |
| None | 4294/9243 (46.5) | ref | ref |  |
| Not regularly | 8/21 (38.1) | 0.71 (0.28, 1.68) | 0.86 (0.33, 2.16) | 0.757 |
| 1/week | 16/56 (28.6) | 0.46 (0.25, 0.81) | 0.69 (0.37, 1.25) | 0.236 |
| Daily | 30/95 (31.6) | 0.53 (0.34, 0.81) | 0.81 (0.51, 1.27) | 0.377 |
| Voluntary activities |  |  |  |  |
| No | 4327/9339 (46.3) | ref | ref |  |
| Yes | 21/76 (27.6) | 0.44 (0.26, 0.72) | 0.69 (0.40, 1.15) | 0.164 |
| Frequency of voluntary activities |  |  |  |  |
| None | 4327/9339 (46.3) | ref | ref |  |
| Not regularly | 1/4 (25.0) | 0.39 (0.02, 3.02) | 0.86 (0.04, 7.99) | 0.902 |
| 1/week | 2/14 (14.3) | 0.19 (0.03, 0.71) | 0.33 (0.05, 1.26) | 0.153 |
| Daily | 18/58 (31.0) | 0.52 (0.29, 0.90) | 0.78 (0.43, 1.37) | 0.395 |
| Cared for a sick or disabled |  |  |  |  |
| No | 4321/9339 (46.3) | ref | ref |  |
| Yes | 27/76 (35.5) | 0.64 (0.39, 1.02) | 0.75 (0.45, 1.22) | 0.253 |
| Frequency of cared for a sick or disabled |  |  |  |  |
| None | 4321/9339 (46.3) | ref | ref |  |
| Not regularly | 8/16 (50.0) | 1.16 (0.43, 3.16) | 1.48 (0.51, 4.31) | 0.464 |
| 1/week | 11/22 (50.0) | 1.16 (0.50, 2.71) | 1.45 (0.59, 3.52) | 0.412 |
| Daily | 8/38 (21.1) | 0.31 (0.13, 0.64) | 0.35 (0.15, 0.74) | 0.010 |
| Attend an educational or training course |  |  |  |  |
| No | 4337/9375 (46.3） | ref | ref |  |
| Yes | 11/40 （27.5） | 0.44 (0.83, 0.90) | 0.90 (0.42, 1.82) | 0.777 |
| Frequency of attend an educational or training course |  |  |  |  |
| None | 4337/9375 (46.3) | ref | ref |  |
| Not regularly | 1/21 (50.0) | 1.16 (0.05, 29.38) | 3.33 (0.13, 85.21) | 0.398 |
| 1/week | 2/9 (22.2) | 0.33 (0.05, 1.37) | 0.49 (0.07, 2.17) | 0.393 |
| Daily | 8/29 (27.6) | 0.44 (0.18, 0.96) | 0.98 (0.40, 2.23) | 0.967 |
| Stock investment |  |  |  |  |
| No | 4327/9348 (46.3) | ref | ref |  |
| Yes | 21/67 (31.3) | 0.53 (0.31, 0.88) | 1.01 (0.58, 1.71) | 0.977 |
| Frequency of stock investment |  |  |  |  |
| None | 4327/9348 (46.3) | ref | ref |  |
| Not regularly | 9/34 (26.5) | 0.42 (0.18, 0.86) | 0.78 (0.34, 1.68) | 0.550 |
| 1/week | 5/10 (50.0) | 1.16 (0.32, 4.17) | 2.34 (0.63, 8.69) | 0.192 |
| Daily | 7/23 (30.4) | 0.51 (0.19, 1.19) | 0.95 (0.36, 2.29) | 0.911 |
| Internet inclusion |  |  |  |  |
| No | 4268/9087 (47.0) | ref | ref |  |
| Yes | 80/328 (24.4) | 0.36 (0.28, 0.47) | 0.67 (0.51, 0.89) | <0.001 |
| Frequency of internet inclusion |  |  |  |  |
| None | 4268/9087 (47.0) | ref | ref |  |
| Not regularly | 53/220 (24.1) | 0.36 (0.26, 0.49) | 0.67 (0.48, 0.93) | 0.019 |
| 1/week | 9/55 (16.4) | 0.22 (0.10, 0.43) | 0.41 (0.18, 0.81) | 0.016 |
| Daily | 18/53 (34.0) | 0.58 (0.32, 1.01) | 1.02 (0.55, 1.82) | 0.953 |
| **Different social activities pattern** |  |  |  |  |
| Pattern 1: Social isolation | 2239/4429 (50.6) | ref | ref |  |
| Pattern 2: Daily interact with friends | 636/1384 (46.0) | 0.83 (0.74, 0.94) | 0.79 (0.70, 0.89) | <0.001 |
| Pattern 3: Occasionally interact with friends and attend team sports (i.e., square dance, Tai Chi) | 438/945 (46.3) | 0.84 (0.73, 0.97) | 0.87 (0.75, 1.01) | 0.061 |
| Pattern 4: Every week interact with friends and attend team sports (i.e., square dance, Tai Chi) | 426/922 (46.2) | 0.84 (0.73, 0.97) | 0.87 (0.75, 1.01) | 0.075 |
| Pattern 5: Diversity social (interact with friends, team sports, recreational [i.e., Mah-jong, played chess or cards], help others) | 528/1426 (37.0) | 0.58 (0.51, 0.65) | 0.70 (0.62, 0.80) | <0.001 |
| Pattern 6: Surf the internet and interact with friends | 81/309 (26.2) | 0.35 (0.27, 0.45) | 0.60 (0.45, 0.79) | <0.001 |

OR, odds ratio; CI, confidence interval.

Logistic regression adjusted for confounding clarified by Directed Acyclic Graphs was used to estimate ORs and 95% CIs.

## Table S6. Risk of different depression trajectories among individuals with different social participation and major pattern (identified by k-means) compared with that of individuals without such conditions.

| **Social activities** | **Stable low symptoms** | **Slight increase symptoms** | | | | **Gradual worsening symptoms** | | | | **Sharp worsening and persist symptoms** | | | |
| --- | --- | --- | --- | --- | --- | --- | --- | --- | --- | --- | --- | --- | --- |
|  | **No. of cases/ total (%)** | **No. of cases/ total (%)** | **Crude OR (95% CI)** | **Adjusted OR (95% CI)** | **p** | **No. of cases/ total (%)** | **Crude OR (95% CI)** | **Adjusted OR (95% CI)** | **p** | **No. of cases/ total (%)** | **Crude OR (95% CI)** | **Adjusted OR (95% CI)** | **p** |
| ***Individual activities*** |  |  |  |  |  |  |  |  |  |  |  |  |  |
| Interact with friends |  |  |  |  |  |  |  |  |  |  |  |  |  |
| No | 3064/5988 (51.2) | 1867/5988 (31.2) | ref | ref |  | 387/5988 (12.6) | ref | ref |  | 670/5988 (21.9) | ref | ref |  |
| Yes | 1788/3427 (52.2) | 1053/3427 (30.7) | 0.97 (0.88, 1.06) | 0.96 (0.87, 1.06) | 0.421 | 236/3427 (13.2) | 1.05 (0.88, 1.24) | 1.02 (0.86, 1.22) | 0.824 | 350/3427 (19.6) | 0.90 (0.88, 1.24) | 0.91 (0.78, 1.05) | 0.205 |
| Frequency of interact with friends |  |  |  |  |  |  |  |  |  |  |  |  |  |
| None | 3064/5988 (51.2) | 1867/5988 (31.2) | ref | ref |  | 387/5988 (12.6) | ref | ref |  | 670/5988 (21.9) | ref | ref |  |
| Not regularly | 890/1692 (52.6) | 509/1692 (30.1) | 0.94 (0.83, 1.06) | 0.90 (0.79, 1.02) | 0.099 | 116/1692 (13.0) | 1.03 (0.83, 1.29) | 0.94 (0.75, 1.18) | 0.594 | 177/1692 (19.9) | 0.91 (0.76, 1.09) | 0.87 (0.71, 1.05) | 0.142 |
| 1/week | 405/773 (52.4) | 249/773 (32.2) | 1.01 (0.85, 1.19) | 1.06 (0.89, 1.26) | 0.494 | 43/773 (10.6) | 0.84 (0.60, 1.17) | 0.91 (0.65, 1.27) | 0.561 | 76/773 (18.8) | 0.86 (0.66, 1.11) | 0.98 (0.75, 1.29) | 0.890 |
| Daily | 493/962 (51.2) | 295/962 (30.7) | 0.98 (0.84, 1.15) | 0.99 (0.85, 1.16) | 0.929 | 77/962 (15.6) | 1.24 (0.95, 1.61) | 1.25 (0.95, 1.63) | 0.106 | 97/962 (19.7) | 0.90 (0.71, 1.14) | 0.92 (0.72, 1.18) | 0.534 |
| Recreational (mah-jong, cards, chess, etc.) |  |  |  |  |  |  |  |  |  |  |  |  |  |
| No | 3650/7383 (49.4） | 2365/7383 (32.0) | ref | ref |  | 507/7383 (13.9) | ref | ref |  | 861/7383 (23.6) | ref | ref |  |
| Yes | 1202/2032 (59.2) | 555/2032 (27.3) | 0.71 (0.64, 0.80) | 0.78 (0.70, 0.88) | <0.001 | 116/2032 (9.7) | 0.69 (0.56, 0.86) | 0.80 (0.64, 0.99) | <0.001 | 159/2032 (13.2) | 0.56 (0.47, 0.67) | 0.74 (0.61, 0.89) | <0.001 |
| Frequency of recreational |  |  |  |  |  |  |  |  |  |  |  |  |  |
| None | 3650/7383 (49.4) | 2365/7383 (32.0) | ref | ref |  | 507/7383 (13.9) | ref | ref |  | 861/7383 (23.6) | ref | ref |  |
| Not regularly | 339/538 (63.0) | 139/538 (25.8) | 0.63 (0.52, 0.78) | 0.70 (0.57, 0.87) | <0.001 | 24/538 (7.1) | 0.51 (0.33, 0.78) | 0.60 (0.39, 0.92) | 0.02 | 36/538 (10.6) | 0.45 (0.32, 0.64) | 0.56 (0.39, 0.81) | 0.002 |
| 1/week | 422/695 (60.7) | 189/695 (27.2) | 0.69 (0.58, 0.83) | 0.76 (0.63, 0.91) | 0.003 | 39/695 (9.2) | 0.67 (0.47, 0.94) | 0.77 (0.54, 1.09) | 0.138 | 45/695 (10.7) | 0.45 (0.33, 0.62) | 0.60 (0.43, 0.83) | 0.002 |
| Daily | 441/799 (55.2) | 227/799 (28.4) | 0.79 (0.67, 0.94) | 0.86 (0.73, 1.03) | 0.097 | 53/799 (12.0) | 0.87 (0.64, 1.17) | 0.96 (0.71, 1.31) | 0.82 | 78/799 (17.7) | 0.75 (0.58, 0.96) | 1.01 (0.77, 1.31) | 0.965 |
| Provide help to others |  |  |  |  |  |  |  |  |  |  |  |  |  |
| No | 4454/8729 (51.0) | 2730/8729 (31.3) | ref | ref |  | 581/8729 (13.0) | ref | ref |  | 964/8729 (21.6) | ref | ref |  |
| Yes | 398/686 (58.0) | 190/686 (27.7) | 0.78 (0.65, 0.93) | 0.86 (0.72, 1.04) | 0.114 | 42/686 (10.6) | 0.81 (0.65, 0.93) | 0.91 (0.65, 1.27) | 0.577 | 56/686 (14.1) | 0.65 (0.49, 0.87) | 0.84 (0.62, 1.14) | 0.267 |
| Frequency of provide help to others |  |  |  |  |  |  |  |  |  |  |  |  |  |
| None | 4454/8729 (51.0) | 2730/8729 (31.3) | ref | ref |  | 581/8729 (13.0) | ref | ref |  | 964/8729 (21.6) | ref | ref |  |
| Not regularly | 41/69 (59.4) | 14/69 (20.3) | 0.56 (0.30, 1.02) | 0.60 (0.32, 1.13) | 0.113 | 6/69 (14.6) | 1.12 (0.47, 2.65) | 1.22 (0.50, 2.94) | 0.653 | 8/69 (19.5) | 0.90 (0.42, 1.93) | 1.05 (0.47, 2.33) | 0.906 |
| 1/week | 77/122 (63.1) | 31/122 (25.4) | 0.66 (0.43, 1.00) | 0.83 (0.54, 1.27) | 0.387 | 5/122 (6.5) | 0.50 (0.20, 1.24) | 0.66 (0.26, 1.66) | 0.375 | 9/122 (11.7) | 0.54 (0.27, 1.09) | 0.92 (0.45, 1.88) | 0.822 |
| Daily | 280/495 (56.6) | 145/495 (29.3) | 0.84 (0.69, 1.04) | 0.91 (0.73, 1.12) | 0.371 | 31/495 (11.1) | 0.85 (0.58, 1.24) | 0.92 (0.62, 1.36) | 0.672 | 39/495 (13.9) | 0.64 (0.46, 0.91) | 0.80 (0.56, 1.14) | 0.219 |
| Attend team sports |  |  |  |  |  |  |  |  |  |  |  |  |  |
| No | 4358/8676 (50.2) | 2743/8676 (31.6) | ref | ref |  | 594/8676 (13.6) | ref | ref |  | 981/8676 (22.5) | ref | ref |  |
| Yes | 494/739 (66.8) | 177/739 (24.0) | 0.57 (0.48, 0.68) | 0.78 (0.65, 0.95) | 0.013 | 29/739 (5.9) | 0.43 (0.29, 0.63) | 0.67 (0.45, 1.01) | 0.055 | 39/739 (7.9) | 0.35 (0.25, 0.49) | 0.64 (0.45, 0.91) | 0.014 |
| Frequency of attend team sports |  |  |  |  |  |  |  |  |  |  |  |  |  |
| None | 4358/8676 (50.2) | 2743/8676 (31.6) | ref | ref |  | 594/8676 (13.6) | ref | ref |  | 981/8676 (22.5) | ref | ref |  |
| Not regularly | 327/488 (67.0) | 118/488 (24.2) | 0.57 (0.46, 0.71) | 0.80 (0.64, 1.00) | 0.053 | 18/488 (5.5) | 0.40 (0.25, 0.65) | 0.64 (0.39, 1.05) | 0.076 | 25/488 (7.6) | 0.34 (0.22, 0.51) | 0.62 (0.40, 0.96) | 0.033 |
| 1/week | 100/148 (67.6) | 34/148 (23.0) | 0.54 (0.36, 0.80) | 0.74 (0.50, 1.11) | 0.151 | 6/148 (6.0) | 0.44 (0.19, 1.01) | 0.71 (0.30, 1.65) | 0.424 | 8/148 (8.0) | 0.36 (0.17, 0.73) | 0.66 (0.31, 1.39) | 0.274 |
| Daily | 67/103 (65.0) | 25/103 (24.3) | 0.59 (0.37, 0.94) | 0.78 (0.48, 1.25) | 0.296 | 5/103 (7.5) | 0.55 (0.22, 1.36) | 0.80 (0.32, 2.03) | 0.638 | 6/103 (9.0) | 0.40 (0.17, 0.92) | 0.68 (0.28, 1.61) | 0.375 |
| Community organization |  |  |  |  |  |  |  |  |  |  |  |  |  |
| No | 4733/9243 (51.2) | 2878/9243 (31.1) | ref | ref |  | 621/9243 (13.1) | ref | ref |  | 1011/9243 (21.4) | ref | ref |  |
| Yes | 119/172 (69.2) | 42/172 (24.4) | 0.58 (0.41, 0.83) | 0.79 (0.55, 1.14) | 0.202 | 2/172 (1.7) | 0.13 (0.03, 0.52) | 0.19 (0.05, 0.79) | 0.022 | 9/172 (7.6) | 0.35 (0.18, 0.70) | 0.57 (0.28, 1.15) | 0.117 |
| Frequency of community organization |  |  |  |  |  |  |  |  |  |  |  |  |  |
| None | 4733/9243 (51.2) | 2878/9243 (31.1) | ref | ref |  | 621/9243 (13.1) | ref | ref |  | 1011/9243 (21.4) | ref | ref |  |
| Not regularly | 12/21 (57.1) | 6/21 (28.6) | 0.82 (0.31, 2.19) | 1.03 (0.38, 2.83) | 0.953 |  | Inf | Inf |  | 3/21 (25.0) | 1.17 (0.33, 4.16) | 1.34 (0.33, 5.38) | 0.682 |
| 1/week | 41/56 (73.2) | 15/56 (26.8) | 0.60 (0.33, 1.09) | 0.80 (0.43, 1.47) | 0.474 |  | Inf | Inf |  |  | Inf | Inf |  |
| Daily | 66/95 (69.5) | 21/95 (22.1) | 0.52 (0.32, 0.86) | 0.74 (0.44, 1.22) | 0.236 | 2/95 (3.0) | 0.23 (0.06, 0.94) | 0.37 (0.9, 1.54) | 0.174 | 6/95 (9.1） | 0.43 (0.18, 0.98) | 0.75 (0.31, 1.82) | 0.535 |
| Voluntary activities |  |  |  |  |  |  |  |  |  |  |  |  |  |
| No | 4797/9339 (51.4) | 2903/9339 (31.1) | ref | ref |  | 623/9339 (13.0) | ref | ref |  | 1016/9339 (21.2) | ref | ref |  |
| Yes | 55/76 (72.4) | 17/76 (22.4) | 0.51 (0.30, 0.88) | 0.75 (0.43, 1.32) | 0.323 |  | Inf | Inf |  | 4/76 (7.3) | 0.34 (0.12, 0.94) | 0.76 (0.27, 2.15) | 0.604 |
| Frequency of voluntary activities |  |  |  |  |  |  |  |  |  |  |  |  |  |
| None | 4797/9339 (51.4) | 2903/9339 (31.1) | ref | ref |  | 623/9339 (13.0) | ref | ref |  | 1016/9339 (21.2) | ref | ref |  |
| Not regularly | 3/4 (75.0) |  | Inf | Inf |  |  | Inf | Inf |  | 1/4 (33.3) | 1.57 (0.16, 15.1) | 5.27 (0.37, 74.34) | 0.219 |
| 1/week | 11/14 (78.6) | 3/14 (21.4) | 0.45 (0.13, 1.62) | 0.65 (0.17, 2.44) | 0.524 |  | Inf | Inf |  |  | Inf | Inf |  |
| Daily | 41/58 (70.7) | 14/58 (24.1) | 0.56 (0.31, 1.04) | 0.82 (0.44, 1.52) | 0.523 |  | Inf | Inf |  | 3/58 (7.3) | 0.35 (0.11, 1.12) | 0.75 (0.23, 2.48) | 0.641 |
| Cared for a sick or disabled |  |  |  |  |  |  |  |  |  |  |  |  |  |
| No | 4801/9339 (51.4) | 2903/9339 (31.1) | ref | ref |  | 621/9339 (12.9) | ref | ref |  | 1014/9339 (21.1) | ref | ref |  |
| Yes | 51/76 (67.1) | 17/76 (22.4) | 0.55 (0.32, 0.96) | 0.64 (0.36, 1.13） | 0.122 | 2/76 (3.9) | 0.30 (0.07, 1.25) | 0.36 (0.09, 1.50) | 0.160 | 6/76 (11.8) | 0.56 (0.24, 1.30) | 0.74 (0.31, 1.80) | 0.507 |
| Frequency of cared for a sick or disabled |  |  |  |  |  |  |  |  |  |  |  |  |  |
| None | 4801/9339 (51.4) | 2903/9339 (21.1) | ref | ref |  | 621/9339 (12.9) | ref | ref |  | 1014/9339 (21.1) | ref | ref |  |
| Not regularly | 9/16 (56.3) | 5/16 (31.3) | 0.92 (0.31, 2.74) | 1.13 (0.36, 3.54) | 0.835 |  | Inf | Inf |  | 2/16 (22.2) | 1.05 (0.23, 4.88) | 1.36 (0.26, 7.02) | 0.717 |
| 1/week | 11/22 (50.0) | 7/22 (31.8) | 1.05 (0.41, 2.72) | 1.26 (0.48, 3.35) | 0.637 | 1/22 (9.1) | 0.70 (0.09, 5.45) | 0.90 (0.11, 7.17) | 0.922 | 3/22 (27.3) | 1.29 (0.36, 4.64) | 1.94 (0.50, 7.53) | 0.341 |
| Daily | 31/38 (81.6) | 5/38 (13.2) | 0.27 (0.10, 0.69) | 0.30 (0.11, 0.78) | 0.014 | 1/38 (3.2) | 0.25 (0.03, 1.83) | 0.28 (0.04, 2.08) | 0.213 | 1/38 (3.2) | 0.15 (0.02, 1.12) | 0.19 (0.03, 1.48) | 0.113 |
| Attend an educational or training course |  |  |  |  |  |  |  |  |  |  |  |  |  |
| No | 4824/9375 (51.5) | 2911/9375 (31.1) | ref | ref |  | 623/9375 (12.9) | ref | ref |  | 1017/9375 (21.1) | ref | ref |  |
| Yes | 28/40 (70.0） | 9/40 (22.5) | 0.53 (0.25, 1.13) | 1.02 (0.47, 2.22) | 0.966 |  | Inf | Inf |  | 3/40 (10.7) | 0.52 (0.16, 1.69) | 1.64 (0.47, 5.70) | 0.434 |
| Frequency of attend an educational or training course |  |  |  |  |  |  |  |  |  |  |  |  |  |
| None | 4824/9375 (51.5) | 2911/9375 (31.1） | ref | ref |  | 623/9375 (12.9) | ref | ref |  | 1017/9375 (21.1) | ref | ref |  |
| Not regularly | 2/2 (100.0) |  | Inf | Inf |  |  | Inf | Inf |  |  | Inf | Inf |  |
| 1/week | 6/9 (66.7) | 2/9 (22.2) | 0.55 (0.11, 2.74) | 0.74 (0.14, 3.86) | 0.719 |  | Inf | Inf |  | 1/9 (16.7) | 0.79 (0.10, 6.57) | 1.70 (0.19, 15.34) | 0.635 |
| Daily | 20/29 (69.0) | 7/29 (24.1) | 0.58 (0.24, 1.37) | 1.23 (0.51, 2.99) | 0.649 |  | Inf | Inf |  | 2/29 (10.0) | 0.47 (0.11, 2.03) | 1.73 (0.38, 7.83) | 0.478 |
| Stock investment |  |  |  |  |  |  |  |  |  |  |  |  |  |
| No | 4809/9348 (51.4) | 2903/9348 (31.1) | ref | ref |  | 620/9348 (12.9) | ref | ref |  | 1016/9348 (21.1) | ref | ref |  |
| Yes | 43/67 (64.2) | 17/67 (25.4) | 0.65 (0.37, 1.15) | 1.13 (0.63, 2.02) | 0.687 | 3/67 (7.0) | 0.54 (0.17, 1.75) | 1.24 (0.37, 4.15) | 0.726 | 4/67 (9.3) | 0.44 (0.16, 1.23) | 1.23 (0.42, 3.57) | 0.704 |
| Frequency of stock investment |  |  |  |  |  |  |  |  |  |  |  |  |  |
| None | 4809/9348 (51.4) | 2903/9348 (31.1) | ref | ref |  | 620/9348 (12.9) | ref | ref |  | 1016/9348 (21.1) | ref | ref |  |
| Not regularly | 21/34 (61.8) | 10/34 (29.4) | 0.79 (0.37, 1.68) | 1.35 (0.62, 2.95) | 0.681 | 2/34 (9.5) | 0.74 (0.17, 3.16) | 1.62 (0.36, 7.25) | 0.530 | 1/34 (4.8) | 0.23 (0.03, 1.68) | 0.65 (0.9, 5.0) | 0.681 |
| 1/week | 6/10 (60.0) | 3/10 (30.0） | 0.83 (0.21, 3.31) | 1.44 (0.35, 5.90) | 0.533 |  | Inf | Inf |  | 1/10 (16.7) | 0.79 (0.09, 6.56) | 2.01 (0.22, 17.89) | 0.533 |
| Daily | 16/23 (69.6) | 4/23 (17.4) | 0.41 (0.14, 1.24) | 0.72 (0.24, 2.19) | 0.504 | 1/23 (6.3) | 0.48 (0.06, 3.66) | 1.16 (0.15, 9.03) | 0.885 | 2/23 (12.5) | 0.59 (0.14, 2.58) | 1.68 (0.39, 7.62) | 0.504 |
| Internet inclusion |  |  |  |  |  |  |  |  |  |  |  |  |  |
| No | 4622/9087 (50.9) | 2844/9087 (31.3) | ref | ref |  | 618/9087 (13.4) | ref | ref |  | 1003/9087 (21.7) | ref | ref |  |
| Yes | 230/328 (70.1) | 76/328 (23.2) | 0.54 (0.41, 0.70) | 0.98 (0.73, 1.31) | 0.888 | 5/328 (2.2) | 0.16 (0.07, 0.40) | 0.36 (0.14, 0.90) | 0.028 | 17/328 (7.4) | 0.34 (0.20, 0.56) | 1.00 (0.58, 1.71) | 0.996 |
| Frequency of internet inclusion |  |  |  |  |  |  |  |  |  |  |  |  |  |
| None | 4622/9087 (50.9) | 2844/9087 (31.3) | ref | ref |  | 618/9087 (13.4) | ref | ref |  | 1003/9087 (21.7) | ref | ref |  |
| Not regularly | 154/220 (70.0) | 52/220 (23.6) | 0.55 (0.40, 0.75) | 1.00 (0.71, 1.41) | 0.997 | 4/220 (2.6） | 0.19 (0.07, 0.53) | 0.43 (0.16, 1.21) | 0.110 | 10/220 (6.5) | 0.30 (0.16, 0.57) | 0.86 (0.43, 1.70) | 0.655 |
| 1/week | 43/55 (78.2) | 9/55 (16.4) | 0.34 (0.17, 0.70) | 0.62 (0.29, 1.29) | 0.201 | 1/55 (2.3) | 0.17 (0.02, 1.27） | 0.39 (0.05, 2.87) | 0.353 | 2/55 (4.7) | 0.21 (0.05, 0.89) | 0.66 (0.15, 2.80) | 0.568 |
| Daily | 33/53 (62.3) | 15/53 (28.3) | 0.74 (0.40, 1.36) | 1.32 (0.70, 2.48) | 0.388 |  | Inf | Inf |  | 5/53 (15.2) | 0.70 (0.27, 1.79) | 2.03 (0.76, 5.46) | 0.158 |
| ***Different social activities pattern*** | |  |  |  |  |  |  |  |  |  |  |  |  |
| Pattern 1 | 2102/4429 (47.5) | 1454/4429 (32.8) | ref | ref |  | 310/4429 (14.7) | ref | ref |  | 563/4429 (26.8) | ref | ref |  |
| Pattern 2 | 712/1384 (51.5) | 427/1384 (30.9) | 0.87 (0.76, 0.99) | 0.83 (0.72, 0.95) | 0.009 | 96/1384 (13.5) | 0.91 (0.72, 1.17) | 0.85 (0.66, 1.09) | 0.190 | 149/1384 (20.9) | 0.78 (0.64, 0.95) | 0.75 (0.61, 0.93) | 0.008 |
| Pattern 3 | 492/945 (52.1) | 282/1384 (29.8) | 0.83 (0.71, 0.97) | 0.85 (0.72, 1.00) | 0.056 | 72/945 (14.6) | 0.99 (0.75, 1.31) | 1.03 (0.78, 1.36) | 0.836 | 99/945 (20.1) | 0.75 (0.59, 0.95) | 0.83 (0.65, 1.06) | 0.130 |
| Pattern 4 | 472/922 (51.2) | 297/922 (32.2) | 0.91 (0.78, 1.07) | 0.94 (0.80, 1.11) | 0.457 | 64/922 (13.6) | 0.92 (0.69, 1.23) | 0.96 (0.72, 1.28) | 0.775 | 89/922 (18.9) | 0.70 (0.55, 0.90) | 0.81 (0.62, 1.04) | 0.101 |
| Pattern 5 | 863/1426 (60.5) | 385/1426 (27.0) | 0.64 (0.56, 0.74) | 0.76 (0.66, 0.87) | <0.001 | 75/1426 (8.7) | 0.59 (0.45, 0.77) | 0.73 (0.56, 0.95) | 0.022 | 103/1426 (11.9) | 0.45 (0.36, 0.56) | 0.63 (0.50, 0.80) | <0.001 |
| Pattern 6 | 211/309 (68.3) | 75/309 (24.3) | 0.51 (0.39, 0.67) | 0.86 (0.64, 1.16) | 0.329 | 6/309 (2.8) | 0.19 (0.08, 0.44) | 0.39 (0.17, 0.92) | 0.030 | 17/309 (8.1) | 0.30 (0.18, 0.50) | 0.78 (0.45, 1.35) | 0.380 |

OR, odds ratio; CI, confidence interval.

Multinomial logistic regression adjusted for confounding clarified by Directed Acyclic Graphs was used to estimate ORs and 95% CIs.

## Table S7. Risk of new onset depression among individuals with different social participation and major pattern (identified by k-means) compared with that of individuals without such conditions by excluding patients missing baseline depression status.

| **Social activities** | **No. of cases/ total (%)** | **Crude OR (95% CI)** | **Adjusted OR (95% CI)** | **p** |
| --- | --- | --- | --- | --- |
| ***Individual activities*** |  |  |  |  |
| Interact with friends |  |  |  |  |
| No | 2477/5465 (45.3) | ref | ref |  |
| Yes | 1405/3200 (43.9) | 0.94 (0.86, 1.03) | 0.91 (0.83, 1.00) | 0.044 |
| Frequency of interact with friends |  |  |  |  |
| None | 2477/5465 (45.3) | ref | ref |  |
| Not regularly | 702/1592 (44.1) | 0.95 (0.85, 1.06) | 0.87 (0.78, 0.98) | 0.023 |
| 1/week | 324/722 (44.9) | 0.98 (0.84, 1.15) | 1.02 (0.87, 1.20) | 0.795 |
| Daily | 379/886 (42.8) | 0.90 (0.78, 1.04) | 0.89 (0.77, 1.04) | 0.139 |
| Recreational (mah-jong, cards, chess, etc.) |  |  |  |  |
| No | 3146/6745 (46.6) | ref | ref |  |
| Yes | 736/1920 (38.3) | 0.71 (0.64, 0.79) | 0.79 (0.71, 0.88) | <0.001 |
| Frequency of recreational |  |  |  |  |
| None | 3146/6745 (46.6) | ref | ref |  |
| Not regularly | 161/497 (32.4) | 0.55 (0.45, 0.66) | 0.65 (0.53, 0.80) | <0.001 |
| 1/week | 239/662 (36.1） | 0.65 (0.55, 0.76) | 0.72 (0.60, 0.85) | <0.001 |
| Daily | 336/761 (44.2) | 0.90 (0.78, 1.05) | 0.97 (0.83, 1.14) | 0.713 |
| Provide help to others |  |  |  |  |
| No | 3612/8012 (45.1) | ref | ref |  |
| Yes | 270/653 (41.3) | 0.86 (0.73, 1.01) | 0.93 (0.69, 1.11) | 0.428 |
| Frequency of provide help to others |  |  |  |  |
| None | 3612/8012 (45.1) | ref | ref |  |
| Not regularly | 26/64 (40.6) | 0.83 (0.50, 1.37) | 0.91 (0.53, 1.52) | 0.709 |
| 1/week | 38/118 (32.2) | 0.58 (0.39, 0.85) | 0.70 (0.46, 1.04) | 0.086 |
| Daily | 206/471 (43.7) | 0.95 (0.78, 1.14) | 1.00 (0.82, 1.22) | 0.989 |
| Attend team sports |  |  |  |  |
| No | 3673/7945 (46.2) | ref | ref |  |
| Yes | 209/720 (29.0) | 0.48 (0.40, 0.56) | 0.70 (0.58, 0.84) | <0.001 |
| Frequency of attend team sports |  |  |  |  |
| None | 3673/7945 (46.2) | ref | ref |  |
| Not regularly | 137/477 (28.7) | 0.47 (0.38, 0.57) | 0.70 (0.56, 0.86) | 0.001 |
| 1/week | 42/144 (29.2) | 0.48 (0.33, 0.68) | 0.70 (0.48, 1.01) | 0.065 |
| Daily | 30/99 (30.3) | 0.51 (0.32, 0.77) | 0.71 (0.45, 1.10) | 0.133 |
| Community organization |  |  |  |  |
| No | 3832/8501 (45.1) | ref | ref |  |
| Yes | 50/164 (30.5) | 0.53 (0.38, 0.74) | 0.80 (0.56, 1.13) | 0.217 |
| Frequency of community organization |  |  |  |  |
| None | 3832/8501 (45.1) | ref | ref |  |
| Not regularly | 8/21 (38.1) | 0.75 (0.30, 1.78) | 0.88 (0.33, 2.20) | 0.784 |
| 1/week | 14/54 (25.9) | 0.43 (0.22, 0.77) | 0.63 (0.32, 1.16) | 0.153 |
| Daily | 28/89 (31.5) | 0.56 (0.35, 0.87) | 0.90 (0.56, 1.42) | 0.661 |
| Voluntary activities |  |  |  |  |
| No | 3861/8590 (44.9) | ref | ref |  |
| Yes | 21/75 (28.0) | 0.48 (0.28, 0.78) | 0.72 (0.41, 1.19) | 0.213 |
| Frequency of voluntary activities |  |  |  |  |
| None | 3861/8590 (44.9) | ref | ref |  |
| Not regularly | 1/4 (25.0) | 0.41 (0.02, 3.19) | 0.85 (0.04, 7.95) | 0.895 |
| 1/week | 2/14 (14.3) | 0.20 (0.03, 0.75) | 0.34 (0.05, 1.31) | 0.169 |
| Daily | 18/57 (31.6) | 0.57 (0.32, 0.97) | 0.81 (0.44, 1.43) | 0.478 |
| Cared for a sick or disabled |  |  |  |  |
| No | 3856/8591 (44.9) | ref | ref |  |
| Yes | 26/74 (35.1) | 0.67 (0.41, 1.06) | 0.77 (0.46, 1.26) | 0.306 |
| Frequency of cared for a sick or disabled |  |  |  |  |
| None | 3856/8591 (44.9) | ref | ref |  |
| Not regularly | 7/15 (46.7) | 1.07 (0.38, 3.00) | 1.44 (0.48, 4.25) | 0.507 |
| 1/week | 11/22 (50.0) | 1.23 (0.53, 2.87) | 1.45 (0.59, 3.55) | 0.410 |
| Daily | 8/37 (21.6) | 0.34 (0.14, 0.71) | 0.38 (0.16, 0.80) | 0.017 |
| Attend an educational or training course |  |  |  |  |
| No | 3871/8626 (44.9) | ref | ref |  |
| Yes | 11/39 (28.2) | 0.48 (0.23, 0.94) | 0.98 (0.45, 2.00) | 0.964 |
| Frequency of attend an educational or training course |  |  |  |  |
| None | 3871/8626 (44.9） | ref | ref |  |
| Not regularly | 1/2 (50.0) | 1.23 (0.05, 31.07) | 3.59 (0.14, 92.10) | 0.369 |
| 1/week | 2/8 (25.0) | 0.41 (0.06, 1.78) | 0.64 (0.09, 2.99) | 0.600 |
| Daily | 8/29 (27.6) | 0.47 (0.19, 1.02) | 1.00 (0.40, 2.28) | 0.994 |
| Stock investment |  |  |  |  |
| No | 3862/8600 (44.9) | ref | ref |  |
| Yes | 20/65 (30.8) | 0.55 (0.31, 0.91) | 1.01 (0.57, 1.73) | 0.975 |
| Frequency of stock investment |  |  |  |  |
| None | 3862/8600 (44.9) | ref | ref |  |
| Not regularly | 9/34 (26.5) | 0.44 (0.19, 0.91) | 0.79 (0.34, 1.70) | 0.568 |
| 1/week | 4/9 (44.4) | 0.98 (0.24, 3.71) | 1.94 (0.47, 7.61) | 0.334 |
| Daily | 7/22 (31.8) | 0.57 (0.22, 1.36) | 1.06 (0.40, 2.60) | 0.895 |
| Internet inclusion |  |  |  |  |
| No | 3805/8346 (45.6) | ref | ref |  |
| Yes | 77/319 (24.1) | 0.38 (0.29, 0.49) | 0.69 (0.52, 0.91) | 0.010 |
| Frequency of internet inclusion |  |  |  |  |
| None | 3805/8346 (45.6) | ref | ref |  |
| Not regularly | 52/216 (24.1) | 0.38 (0.27, 0.51) | 0.69 (0.49, 0.96) | 0.032 |
| 1/week | 8/52 (15.4) | 0.22 (0.09, 0.44) | 0.39 (0.17, 0.81) | 0.019 |
| Daily | 17/51 (33.3) | 0.60 (0.33, 1.05) | 1.03 (0.55, 1.87) | 0.916 |
| ***Different social activities pattern*** |  |  |  |  |
| Pattern 1: Social isolation | 1955/3991 (49.0) | ref | ref |  |
| Pattern 2: Daily interact with friends | 590/1301 (45.3) | 0.86 (0.76, 0.98) | 0.80 (0.70, 0.91) | 0.001 |
| Pattern 3: Occasionally interact with friends and attend team sports (i.e., square dance, Tai Chi) | 382/864 (44.2) | 0.83 (0.71, 0.96) | 0.84 (0.72, 0.98) | 0.028 |
| Pattern 4: Every week interact with friends and attend team sports (i.e., square dance, Tai Chi) | 392/862 (45.5) | 0.87 (0.75, 1.01) | 0.88 (0.76, 1.03) | 0.116 |
| Pattern 5: Diversity social (interact with friends, team sports, recreational [i.e., Mah-jong, played chess or cards], help others) | 485/11348 (36.1) | 0.59 (0.52, 0.67) | 0.71 (0.62, 0.81) | <0.001 |
| Pattern 6: Surf the internet and interact with friends | 77/299 (25.8) | 0.36 (0.28, 0.47) | 0.61 (0.46, 0.81) | 0.001 |

OR, odds ratio; CI, confidence interval.

Logistic regression adjusted for confounding clarified by Directed Acyclic Graphs was used to estimate ORs and 95% CIs.

## Table S8. Risk of different depression trajectories among individuals with different social participation and major pattern (identified by k-means) compared with that of individuals without such conditions by excluding patients missing baseline depressive status.

| **Social activities** | **Stable low symptoms** | **Slight increase symptoms** | | | | **Gradual worsening symptoms** | | | | **Sharp worsening and persist symptoms** | | | |
| --- | --- | --- | --- | --- | --- | --- | --- | --- | --- | --- | --- | --- | --- |
|  | **No. of cases/ total (%)** | **No. of cases/ total (%)** | **Crude OR (95% CI)** | **Adjusted OR (95% CI)** | **p** | **No. of cases/ total (%)** | **Crude OR (95% CI)** | **Adjusted OR (95% CI)** | **p** | **No. of cases/ total (%)** | **Crude OR (95% CI)** | **Adjusted OR (95% CI)** | **p** |
| ***Individual activities*** |  |  |  |  |  |  |  |  |  |  |  |  |  |
| Interact with friends |  |  |  |  |  |  |  |  |  |  |  |  |  |
| No | 2887/5465 (52.8) | 1696/5465 (31.0) | ref | ref |  | 343/5465 (6.3) | ref | ref |  | 539/5465 (9.9) | ref | ref |  |
| Yes | 1709/3200 (53.4) | 983/3200 (30.7) | 0.98 (0.89, 1.08) | 0.96 (0.87, 1.07) | 0.467 | 224/3200 (7.0) | 1.10 (0.92, 1.32) | 1.07 (0.89, 1.28) | 0.484 | 284/3200 (8.9) | 0.89 (0.76, 1.04) | 0.87 (0.74, 1.02) | 0.095 |
| Frequency of interact with friends |  |  |  |  |  |  |  |  |  |  |  |  |  |
| None | 2887/5465 (52.8) | 1696/5465 (31.0) | ref | ref |  | 343/5465 (6.3) | ref | ref |  | 539/5465 (9.9) | ref | ref |  |
| Not regularly | 852/1592 (53.5) | 477/1592 (30.0) | 0.95 (0.84, 1.08) | 0.90 (0.79, 1.03) | 0.123 | 113/1592 (7.1) | 1.12 (0.89, 1.40) | 1.00 (0.80, 1.27) | 0.968 | 150/1592 (9.4) | 0.94 (0.77, 1.15) | 0.85 (0.70, 1.05) | 0.131 |
| 1/week | 385/722 (53.3) | 238/722 (33.0) | 1.05 (0.89, 1.25) | 1.09 (0.92, 1.31) | 0.317 | 40/722 (5.5) | 0.87 (0.62, 1.23) | 0.94 (0.66, 1.33) | 0.713 | 59/722 (8.2) | 0.82 (0.61, 1.10) | 0.90 (0.67, 1.22) | 0.501 |
| Daily | 472/886 (53.3) | 268/886 (30.2) | 0.97 (0.82, 1.14) | 0.97 (0.82, 1.15) | 0.742 | 71/886 (8.0) | 1.27 (0.96, 1.66) | 1.29 (0.97, 1.70) | 0.075 | 75/886 (8.5) | 0.85 (0.66, 1.10) | 0.88 (0.67, 1.15) | 0.334 |
| Recreational (mah-jong, cards, chess, etc.) |  |  |  |  |  |  |  |  |  |  |  |  |  |
| No | 3439/6745 (51.0) | 2162/6745 (32.1) | ref | ref |  | 460/6745 (6.8) | ref | ref |  | 684/6745 (10.1) | ref | ref |  |
| Yes | 1157/1920 (60.3) | 517/1920 (26.9) | 0.71 (0.63, 0.80) | 0.77 (0.68, 0.87) | <0.001 | 107/1920 (5.6) | 0.69 (0.55, 0.86) | 0.79 (0.63, 0.99) | 0.043 | 139/1920 (7.2) | 0.60 (0.50, 0.73) | 0.75 (0.61, 0.91) | <0.001 |
| Frequency of recreational |  |  |  |  |  |  |  |  |  |  |  |  |  |
| None | 3439/6745 (51.0) | 2162/6745 (32.1） | ref | ref |  | 460/6745 (6.8) | ref | ref |  | 684/6745 (10.1) | ref | ref |  |
| Not regularly | 319/497 (64.2) | 128/497 (25.8) | 0.64 (0.52, 0.79) | 0.71 (0.57, 0.88) | 0.002 | 22/497 (4.4) | 0.52 (0.33, 0.80) | 0.61 (0.39, 0.96) | 0.033 | 28/497 (5.6) | 0.44 (0.30, 0.66) | 0.54 (0.36, 0.82) | 0.003 |
| 1/week | 414/662 (62.5) | 175/662 (26.4) | 0.67 (0.56, 0.81) | 0.72 (0.60, 0.88) | <0.001 | 34/662 (5.1) | 0.61 (0.43, 0.88) | 0.70 (0.49, 1.02) | 0.062 | 39/662 (5.9) | 0.47 (0.34, 0.66) | 0.58 (0.41, 0.82) | 0.002 |
| Daily | 424/761 (55.7) | 214/761 (28.1) | 0.80 (0.68, 0.95) | 0.86 (0.72, 1.03) | 0.095 | 51/761 (6.7) | 0.90 (0.66, 1.22) | 1.01 (0.73, 1.38) | 0.972 | 72/761 (9.5) | 0.85 (0.66, 1.11) | 1.07 (0.81, 1.40) | 0.644 |
| Provide help to others |  |  |  |  |  |  |  |  |  |  |  |  |  |
| No | 4215/8012 (52.6) | 2497/8012 (31.2) | ref | ref |  | 527/8012 (6.6) | ref | ref |  | 773/8012 (9.6) | ref | ref |  |
| Yes | 381/653 (58.3) | 182/653 (27.9) | 0.81 (0.67, 0.97) | 0.88 (0.73, 1.07) | 0.198 | 40/653 (6.1) | 0.84 (0.60, 1.18) | 0.95 (0.67, 1.34) | 0.757 | 50/653 (7.7) | 0.72 (0.53, 0.97) | 0.88 (0.64, 1.21) | 0.433 |
| Frequency of provide help to others |  |  |  |  |  |  |  |  |  |  |  |  |  |
| None | 4215/8012 (52.6) | 2497/8012 (31.2) | ref | ref |  | 527/8012 (6.6) | ref | ref |  | 773/8012 (9.6) | ref | ref |  |
| Not regularly | 38/64 (59.4) | 13/64 (20.3) | 0.58 (0.31, 1.09) | 0.64 (0.34, 1.22) | 0.179 | 6/64 (9.4) | 1.26 (0.53, 3.00) | 1.44 (0.60, 3.50) | 0.416 | 7/64 (10.9) | 1.00 (0.45, 2.26) | 1.16 (0.50, 2.68) | 0.727 |
| 1/week | 75/118 (63.6) | 29/118 (24.6) | 0.65 (0.42, 1.00) | 0.81 (0.52, 1.26) | 0.352 | 5/118 (4.2) | 0.53 (0.21, 1.32) | 0.71 (0.28, 1.80) | 0.475 | 9/118 (7.6) | 0.65 (0.33, 1.31) | 0.99 (0.49, 2.04) | 0.986 |
| Daily | 268/471 (56.9) | 140/471 (29.7) | 0.88 (0.71, 1.09) | 0.93 (0.75, 1.16) | 0.534 | 29/471 (6.2) | 0.87 (0.58, 1.28) | 0.93 (0.62, 1.39) | 0.737 | 34/471 (7.2) | 0.69 (0.48, 1.00) | 0.82 (0.56, 1.19) | 0.294 |
| Attend team sports |  |  |  |  |  |  |  |  |  |  |  |  |  |
| No | 4110/7945 (51.7) | 2512/7945 (31.6) | ref | ref |  | 539/7945 (6.8) | ref | ref |  | 784/7945 (9.9) | ref | ref |  |
| Yes | 486/720 (67.5) | 167/720 (23.2) | 0.56 (0.47, 0.67) | 0.77 (0.63, 0.93) | 0.008 | 28/720 (3.9) | 0.44 (0.30, 0.65) | 0.70 (0.46, 1.05) | 0.085 | 39/720 (5.4) | 0.42 (0.30, 0.59) | 0.70 (0.49, 1.00) | 0.053 |
| Frequency of attend team sports |  |  |  |  |  |  |  |  |  |  |  |  |  |
| None | 4110/7945 (51.7) | 2512/7945 (31.6) | ref | ref |  | 539/7945 (6.8) | ref | ref |  | 784/7945 (9.9) | ref | ref |  |
| Not regularly | 324/477 (67.9) | 110/477 (23.1) | 0.56 (0.44, 0.69) | 0.76 (0.60, 0.97) | 0.024 | 18/477 (3.8) | 0.42 (0.26, 0.69) | 0.67 (0.41, 1.11) | 0.121 | 25/477 (5.2) | 0.40 (0.27, 0.61) | 0.67 (0.44, 1.04) | 0.077 |
| 1/week | 96/144 (66.7) | 34/144 (23.6) | 0.58 (0.39, 0.86) | 0.79 (0.52, 1.18) | 0.247 | 6/144 (4.2) | 0.48 (0.21, 1.09) | 0.77 (0.33, 1.81) | 0.553 | 8/144 (5.6) | 0.44 (0.21, 0.90) | 0.77 (0.34, 1.54) | 0.401 |
| Daily | 66/99 (66.7) | 23/99 (23.2) | 0.57 (0.35, 0.92) | 0.75 (0.46, 1.22) | 0.250 | 4/99 (4.0) | 0.46 (0.17, 1.27) | 0.70 (0.25, 1.96) | 0.500 | 6/99 (6.1) | 0.48 (0.21, 1.10) | 0.70 (0.34, 1.89) | 0.613 |
| Community organization |  |  |  |  |  |  |  |  |  |  |  |  |  |
| No | 4480/8501 (52.7) | 2641/8501 (31.1) | ref | ref |  | 565/8501 (6.6) | ref | ref |  | 815/8501 (9.6) | ref | ref |  |
| Yes | 116/164 (70.7) | 38/164 (23.2) | 0.56 (0.38, 0.80) | 0.76 (0.52, 1.12) | 0.165 | 2/164 (1.2) | 0.14 (0.03, 0.55) | 0.21 (0.05, 0.87) | 0.032 | 8/164 (4.9) | 0.38 (0.18, 0.78) | 0.61 (0.29, 1.29) | 0.199 |
| Frequency of community organization |  |  |  |  |  |  |  |  |  |  |  |  |  |
| None | 4480/8501 (52.7) | 2641/8501 (31.1) | ref | ref |  | 565/8501 (6.6) | ref | ref |  | 815/8501 (9.6) | ref | ref |  |
| Not regularly | 12/21 (57.1) | 6/21 (28.6) | 0.85 (0.32, 2.27) | 1.01 (0.37, 2.79) | 0.981 |  | Inf | Inf |  | 3/21 (14.3) | 1.38 (0.39, 4.88) | 1.35 (0.34, 5.36) | 0.667 |
| 1/week | 40/54 (74.1) | 14/54 (25.9) | 0.59 (0.32, 1.09) | 0.79 (0.42, 1.47) | 0.454 |  | Inf | Inf |  |  | Inf | Inf |  |
| Daily | 64/89 (71.9) | 18/89 (20.2) | 0.48 (0.28, 0.81) | 0.69 (0.40, 1.19) | 0.183 | 2/89 (2.2) | 0.25 (0.06, 1.02) | 0.43 (0.10, 1.77) | 0.241 | 5/89 (5.6) | 0.43 (0.17, 1.07) | 0.85 (0.33, 2.16) | 0.731 |
| Voluntary activities |  |  |  |  |  |  |  |  |  |  |  |  |  |
| No | 4542/8590 (52.9) | 2662/8590 (31.0) | ref | ref |  | 567/8590 (6.6) | ref | ref |  | 819/8590 (9.5) | ref | ref |  |
| Yes | 54/75 (72.0) | 17/75 (22.7) | 0.54 (0.31, 0.93) | 0.77 (0.44, 1.35) | 0.366 |  | Inf | Inf |  | 4/75 (5.3) | 0.41 (0.15, 1.14) | 0.80 (0.28, 2.28) | 0.680 |
| Frequency of voluntary activities |  |  |  |  |  |  |  |  |  |  |  |  |  |
| None | 4542/8590 (52.9) | 2662/8590 (31.0) | ref | ref |  | 567/8590 (6.6) | ref | ref |  | 819/8590 (9.5) | ref | ref |  |
| Not regularly | 3/4 (75.0) |  | Inf | Inf |  |  | Inf | Inf |  | 1/4 (25.0) | 1.85 (0.19, 1.78) | 5.38 (0.38, 75.50) | 0.212 |
| 1/week | 11/14 (78.6) | 3/14 (21.4) | 0.47 (0.13, 1.67) | 0.67 (0.18, 2.50) | 0.555 |  | Inf | Inf |  |  | Inf | Inf |  |
| Daily | 40/57 (70.2) | 14/57 (24.6) | 0.60 (0.32, 1.10) | 0.84 (0.45, 1.57) | 0.578 |  | Inf | Inf |  | 3/57 (5.3) | 0.42 (1.29, 1.35) | 0.79 (0.24, 2.62) | 0.696 |
| Cared for a sick or disabled |  |  |  |  |  |  |  |  |  |  |  |  |  |
| No | 4547/8591 (52.9) | 2662/8591 (31.0) | ref | ref |  | 565/8591 (6.6) | ref | ref |  | 817/8591 (9.5) | ref | ref |  |
| Yes | 49/74 (66.2) | 17/74 (23.0) | 0.59 (0.34, 1.03) | 0.69 (0.39, 1.21) | 0.197 | 2/74 (2.7) | 0.32 (0.08, 1.34) | 0.41 (0.10, 1.69) | 0.216 | 6/74 (8.1) | 0.68 (0.29, 1.59) | 0.86 (0.36, 2.07) | 0.734 |
| Frequency of cared for a sick or disabled |  |  |  |  |  |  |  |  |  |  |  |  |  |
| None | 4547/8591 (52.9) | 2662/8591 (31.0) | ref | ref |  | 565/8591 (6.6） | ref | ref |  | 817/8591 (9.5) | ref | ref |  |
| Not regularly | 8/15 (53.3) | 5/15 (33.3) | 1.07 (0.35, 3.27) | 1.40 (0.44, 4.47) | 0.567 |  | Inf | Inf |  | 2/15(13.3) | 1.39 (0.29, 6.56) | 1.94 (0.38, 9.79) | 0.423 |
| 1/week | 11/22 (50.0) | 7/22 (31.8) | 1.09 (0.42, 2.81) | 1.23 (0.46, 3.27) | 0.681 | 1/22 (4.5) | 0.73 (0.09, 5.68) | 0.93 (0.12, 7.34) | 0.947 | 3/22 (13.6) | 1.52 (0.42, 5.45) | 2.91 (0.49, 7.45) | 0.353 |
| Daily | 30/37 (81.1) | 5/37 (13.5) | 0.28 (0.11, 0.73) | 0.32 (0.12, 0.84) | 0.021 | 1/37 (2.7) | 0.27 (0.04, 1.97) | 0.31 (0.05, 2.32) | 0.256 | 1/37 (2.7) | 0.19 (0.03, 1.36) | 0.23 (0.03, 1.74) | 0.156 |
| Attend an educational or training course |  |  |  |  |  |  |  |  |  |  |  |  |  |
| No | 4569/8626 (53.0) | 2670/8626 (31.0) | ref | ref |  | 567/8626 (6.6) | ref | ref |  | 820/8626 (9.5) | ref | ref |  |
| Yes | 27/39 (69.2) | 9/39 (23.1) | 0.58 (0.27, 1.23) | 1.08 (0.49, 2.37) | 0.846 |  | Inf | Inf |  | 3/39 (7.7) | 0.62 (0.19, 2.05) | 1.87 (0.54, 6.53) | 0.326 |
| Frequency of attend an educational or training course |  |  |  |  |  |  |  |  |  |  |  |  |  |
| None | 4569/8626 (53.0) | 2670/8626 (31.0) | ref | ref |  | 567/8626 (6.6) | ref | ref |  | 820/8626 (820) | ref | ref |  |
| Not regularly | 2/2 (100.0) |  | Inf | Inf |  |  | Inf | Inf |  |  | Inf | Inf |  |
| 1/week | 5/8 (62.5) | 2/8 (25.0) | 0.68 (0.13, 3.53) | 0.96 (0.17, 5.28) | 0.960 |  | Inf | Inf |  | 1/8 (12.5) | 1.11 (0.13, 9.55) | 2.64 (0.28, 25.63) | 0.395 |
| Daily | 20/29 (69.0) | 7/29 (24.1) | 0.60 (0.25, 1.42) | 1.23 (0.50, 2.99) | 0.654 |  | Inf | Inf |  | 2/29 (6.9） | 0.56 (0.13, 2.39) | 1.77 (0.39, 8.05) | 0.460 |
| Stock investment |  |  |  |  |  |  |  |  |  |  |  |  |  |
| No | 4554/8600 (53.0) | 2662/8600 (31.0) | ref | ref |  | 564/8600 (6.6) | ref | ref |  | 820/8600 (9.5) | ref | ref |  |
| Yes | 42/65 (64.6) | 17/65 (26.2) | 0.69 (0.39, 1.22) | 1.18 (0.99, 1.00) | 0.585 | 3/65 (4.6) | 0.58 (0.18, 1.87) | 1.34 (0.40, 4.48) | 0.639 | 3/65 (4.6) | 0.40 (0.12, 1.28) | 0.98 (0.29, 3.26) | 0.972 |
| Frequency of stock investment |  |  |  |  |  |  |  |  |  |  |  |  |  |
| None | 4554/8600 (53.0) | 2662/8600 (31.0) | ref | ref |  | 564/8600 (6.6) | ref | ref |  | 820/8600 (9.5) | ref | ref |  |
| Not regularly | 21/34 (61.8) | 10/34 (29.4) | 0.81 (0.38, 1.73) | 1.36 (0.63, 2.98) | 0.435 | 2/34 (5.9) | 0.77 (0.18, 3.29) | 1.71 (0.38, 7.67) | 0.481 | 1/34 (2.9) | 0.26 (0.04, 1.97) | 0.63 (0.08, 4.82) | 0.654 |
| 1/week | 6/9 (66.7) | 3/9 (33.3) | 0.86 (0.21, 3.42) | 1.50 (0.37, 6.10) | 0.574 |  | Inf | Inf |  |  | Inf | Inf |  |
| Daily | 15/22 (68.2) | 4/22 (18.2) | 0.46 (0.15, 1.38) | 0.79 (0.26, 2.43) | 0.680 | 1/22 (4.5) | 0.54 (0.07, 4.08) | 1.34 (0.17, 1.05) | 0.780 | 2/22 (9.1) | 0.74 (0.17, 3.24) | 1.89 (0.41, 8.62) | 0.411 |
| Internet inclusion |  |  |  |  |  |  |  |  |  |  |  |  |  |
| No | 4372/8346 (52.4) | 2604/8346 (31.2) | ref | ref |  | 562/8346 (6.7) | ref | ref |  | 808/8346 (9.7) | ref | ref |  |
| Yes | 224/319 (70.2) | 75/319 (23.5) | 0.56 (0.43, 0.73) | 1.01 (0.76, 1.36) | 0.926 | 5/319 (1.6) | 0.17 (0.07, 0.42) | 0.39 (0.16, 0.98) | 0.046 | 15/319 (4.7） | 0.36 (0.21, 0.61) | 0.95 (0.54, 1.68) | 0.856 |
| Frequency of internet inclusion |  |  |  |  |  |  |  |  |  |  |  |  |  |
| None | 4372/8346 (52.4) | 2604/8346 (31.2) | ref | ref |  | 562/8346 (6.7) | ref | ref |  | 808/8346 (9.7） | ref | ref |  |
| Not regularly | 216/151 (69.9) | 52/216 (24.1) | 0.58 (0.42, 0.80) | 1.04 (0.74, 1.47) | 0.832 | 4/216 (1.9) | 0.21 (0.08, 0.56) | 0.47 (0.17, 1.31) | 0.146 | 9/216 (4.2) | 0.32 (0.16, 0.63) | 0.80 (0.39, 1.64) | 0.543 |
| 1/week | 41/52 (78.8) | 9/52 (17.3) | 0.37 (0.18, 0.76) | 0.66 (0.32, 1.39) | 0.278 | 1/52 (1.9) | 0.19 (0.03, 1.38) | 0.42 (0.06, 3.17) | 0.404 | 1/52 (1.9) | 0.13 (0.02, 0.96) | 0.38 (0.05, 2.84) | 0.347 |
| Daily | 32/51 (62.7) | 14/51 (27.5) | 0.73 (0.39, 1.38) | 1.32 (0.69, 2.52) | 0.403 |  | Inf | Inf |  | 5/51 (9.8) | 0.85 (0.33, 2.18) | 2.32 (0.87, 6.20) | 0.092 |
| ***Different social activities pattern*** | |  |  |  |  |  |  |  |  |  |  |  |  |
| Pattern 1 | 1961/3991 (49.1) | 1311/3991 (49.1) | ref | ref |  | 272/3991 (6.8) | ref | ref |  | 447/3991 (11.2) | ref | ref |  |
| Pattern 2 | 682/1301 (52.4) | 402/1301 (30.9) | 0.88 (0.77, 1.02) | 0.83 (0.72, 0.96) | 0.012 | 94/1301 (7.2) | 0.99 (0.77, 1.28) | 0.91 (0.70, 1.17) | 0.446 | 123/1301 (9.5) | 0.79 (0.64, 0.98) | 0.72 (0.57, 0.90) | 0.004 |
| Pattern 3 | 469/864 (54.3) | 253/864 (29.3) | 0.81 (0.68, 0.95) | 0.82 (0.69, 0.97)) | 0.024 | 66/864 (7.6) | 1.01 (0.76, 1.35) | 1.06 (0.79, 1.41) | 0.715 | 76/864 (8.8) | 0.71 (0.55, 0.92) | 0.77 (0.58, 1.00) | 0.054 |
| Pattern 4 | 452/862 (52.4) | 278/862 (32.3) | 0.92 (0.78, 1.08) | 0.94 (0.69, 1.11) | 0.438 | 59/862 (6.8) | 0.94 (0.70, 1.27) | 0.98 (0.72, 1.33) | 0.902 | 73/862 (8.5) | 0.71 (0.54, 0.93) | 0.77 (0.58, 1.00) | 0.053 |
| Pattern 5 | 828/1348 (61.4) | 361/1348 (26.8) | 0.65 (0.57, 0.75) | 0.76 (0.66, 0.88) | <0.001 | 70/1348 (5.2) | 0.61 (0.46, 0.80) | 0.76 (0.57, 1.01) | 0.056 | 89/1348 (6.6) | 0.47 (0.37, 0.60) | 0.62 (0.49, 0.80) | <0.001 |
| Pattern 6 | 204/299 (68.2) | 74/299 (24.7) | 0.54 (0.41, 0.71) | 0.90 (0.67, 1.22) | 0.497 | 6/299 (2.0) | 0.21 (0.09, 0.48) | 0.45 (0.19, 1.04) | 0.062 | 15/299 (5.0) | 0.32 (0.19, 0.55) | 0.75 (0.42, 1.34) | 0.332 |

OR, odds ratio; CI, confidence interval.

Multinomial logistic regression adjusted for confounding clarified by Directed Acyclic Graphs was used to estimate ORs and 95% CIs.

## Table S9. During the follow-up period, information on receiving treatment for depression in all new onset depression populations, and stratify by the trajectory of depressive symptoms.

|  | **Total** | **Class 1**  **“Stable low symptoms”** | **Class 2**  **“Slight increase symptoms”** | **Class 3**  **“Gradual increase symptoms”** | **Class 4**  **“Sharp worsening and persist symptoms”** | ***P*** |
| --- | --- | --- | --- | --- | --- | --- |
| Total receiving treatment, No. (%) | 71 (1.6) | 4 (0.9) | 27 (1.2) | 9 (1.4) | 31 (3.0) | <0.001 |
| 2013 | 18 (0.4) | 2 (0.4) | 6 (0.3) | 2 (0.3) | 8 (0.8) | 0.031 |
| 2015 | 28 (0.6) | 0 (0.0) | 12 (0.5) | 2 (0.3) | 14 (1.4) | <0.001 |
| 2018 | 46 (1.1) | 3 (0.7) | 19 (0.8) | 7 (1.1) | 17 (1.7) | <0.001 |
| Total receiving psychiatric or psychological treatment, No. (%) | 24 (0.6) | 4 (0.9) | 8 (0.4) | 2 (0.3) | 10 (1.0) | <0.001 |
| 2013 | 8 (0.2) | 2 (0.4) | 2 (0.1) | 0 (0.0) | 4 (0.4) | 0.008 |
| 2015 | 10 (0.2) | 0 (0.0) | 6 (0.3) | 0 (0.0) | 4 (0.4) | <0.001 |
| 2018 | 10 (0.2) | 2 (0.4) | 2 (0.1) | 2 (0.3) | 4 (0.4) | <0.001 |
| Total taking anti-depressants, No. (%) | 33 (0.8) | 1 (0.2) | 16 (0.7) | 4 (0.6) | 12 (1.2) | <0.001 |
| 2013 | 11 (0.3) | 2 (0.4) | 4 (0.2) | 1 (0.2) | 4 (0.4) | 0.544 |
| 2015 | 12 (0.3) | 0 (0.0) | 7 (0.3) | 1 (0.2) | 4 (0.4) | 0.541 |
| 2018 | 23 (0.5) | 1 (0.2) | 12 (0.5) | 3 (0.5) | 7 (0.7) | 0.723 |
| Total taking tranquilizers or sleeping pills, No. (%) | 49 (1.1) | 2 (0.4) | 19 (0.8) | 5 (0.8) | 23 (2.3) | <0.001 |
| 2013 | 12 (0.3) | 2 (0.4) | 5 (0.2) | 1 (0.2) | 4 (0.4) | 0.008 |
| 2015 | 21 (0.5) | 0 (0.0) | 8 (0.4) | 1 (0.2) | 12 (1.2) | <0.001 |
| 2018 | 27 (0.6) | 2 (0.4) | 11 (0.5) | 4 (0.6) | 10 (1.0) | <0.001 |

## Figure S1. Flowchart of participant selection.


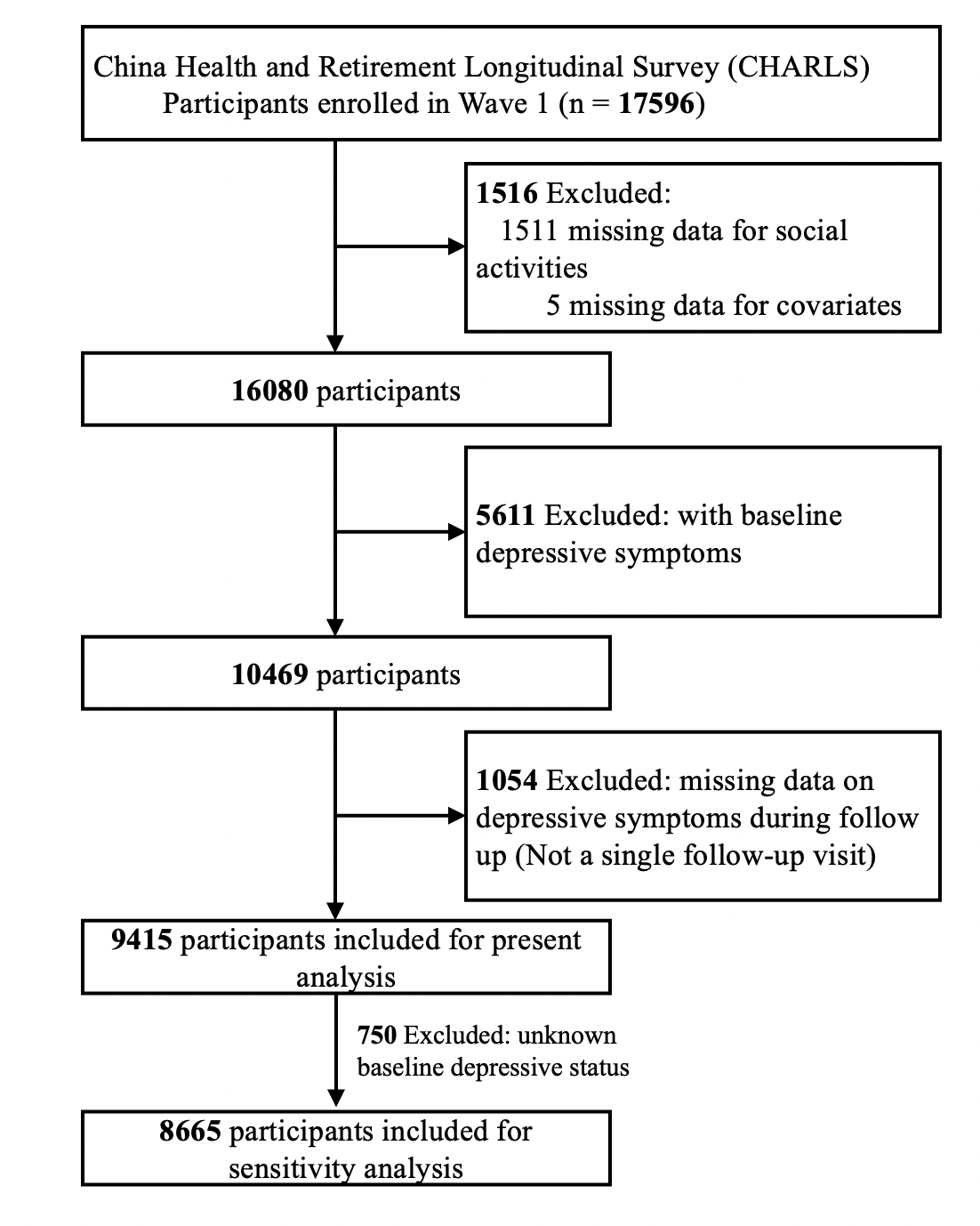


##
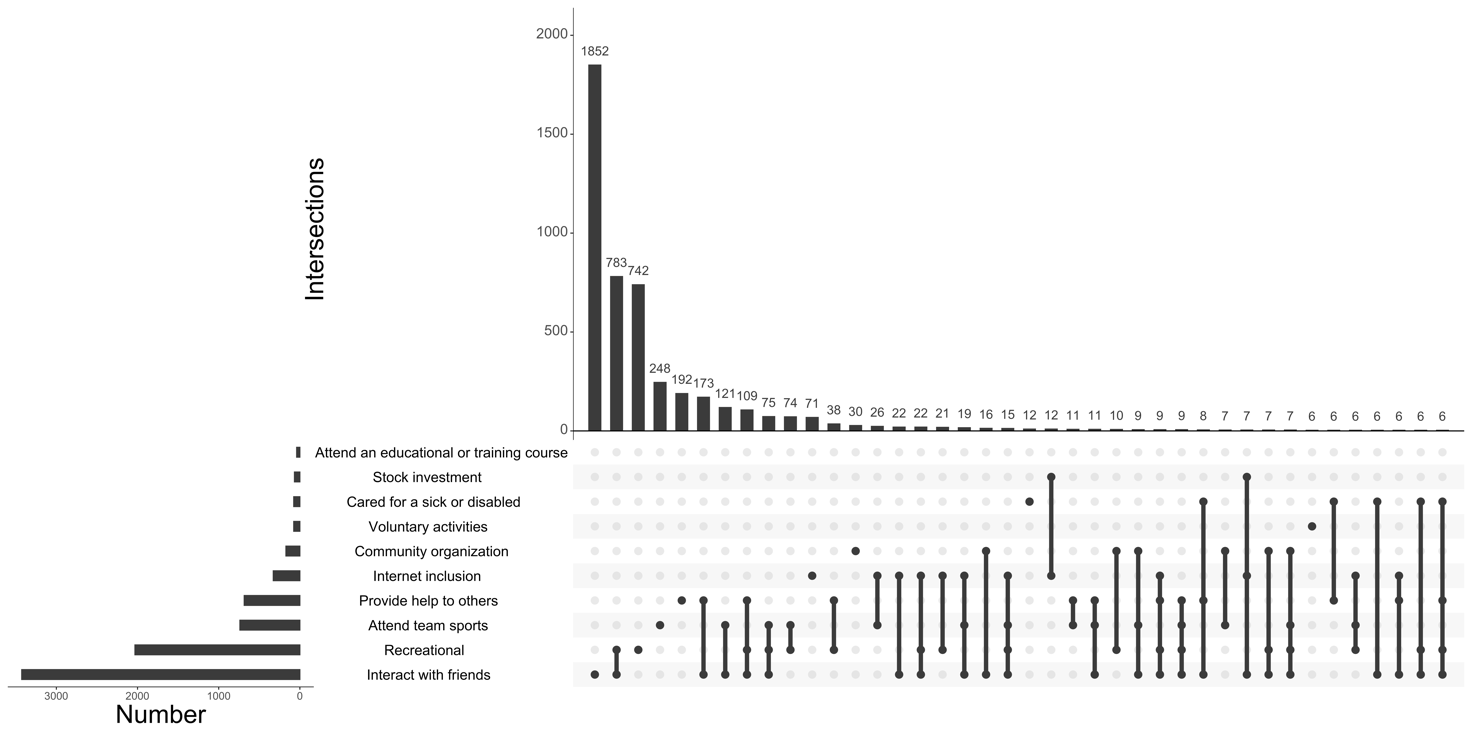
Figure S2. Visualization of intersecting sets of patients with socirty participations.

**Note:** The bar chart above represents the number of patients in each type of group. The bar chart at the bottom left represents the number of events in each type of psychiatric event. The dotted line at the bottom right shows the types of events in the group. One dot indicates that the patient has this characteristic, and two dots mean that the group of patients has two characteristics. The four dots represent the presence of four social participation features.

## Figure S3. The elbow plot is made by calculating the within-cluster sum of squares with k ranging from 1 to 10.


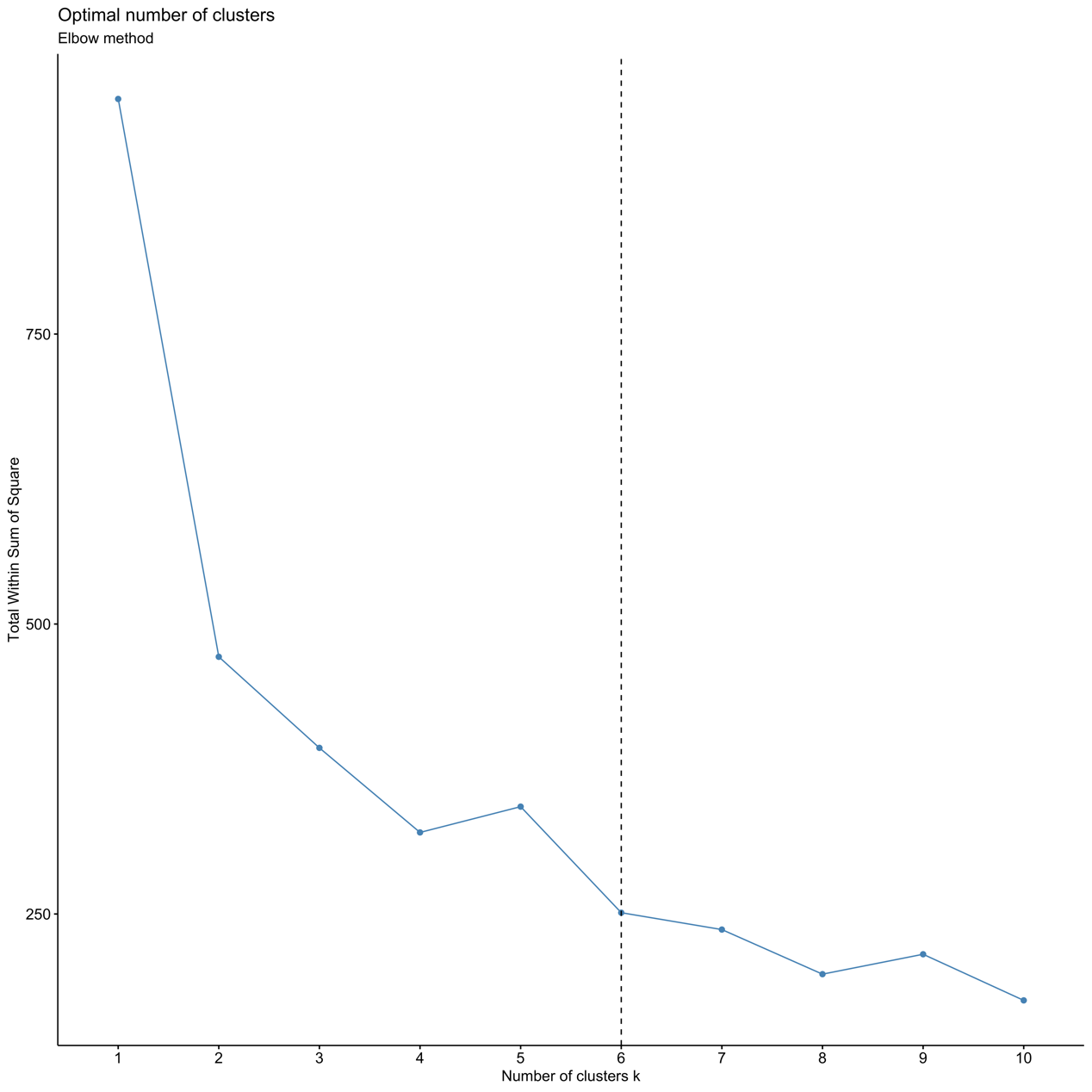


##
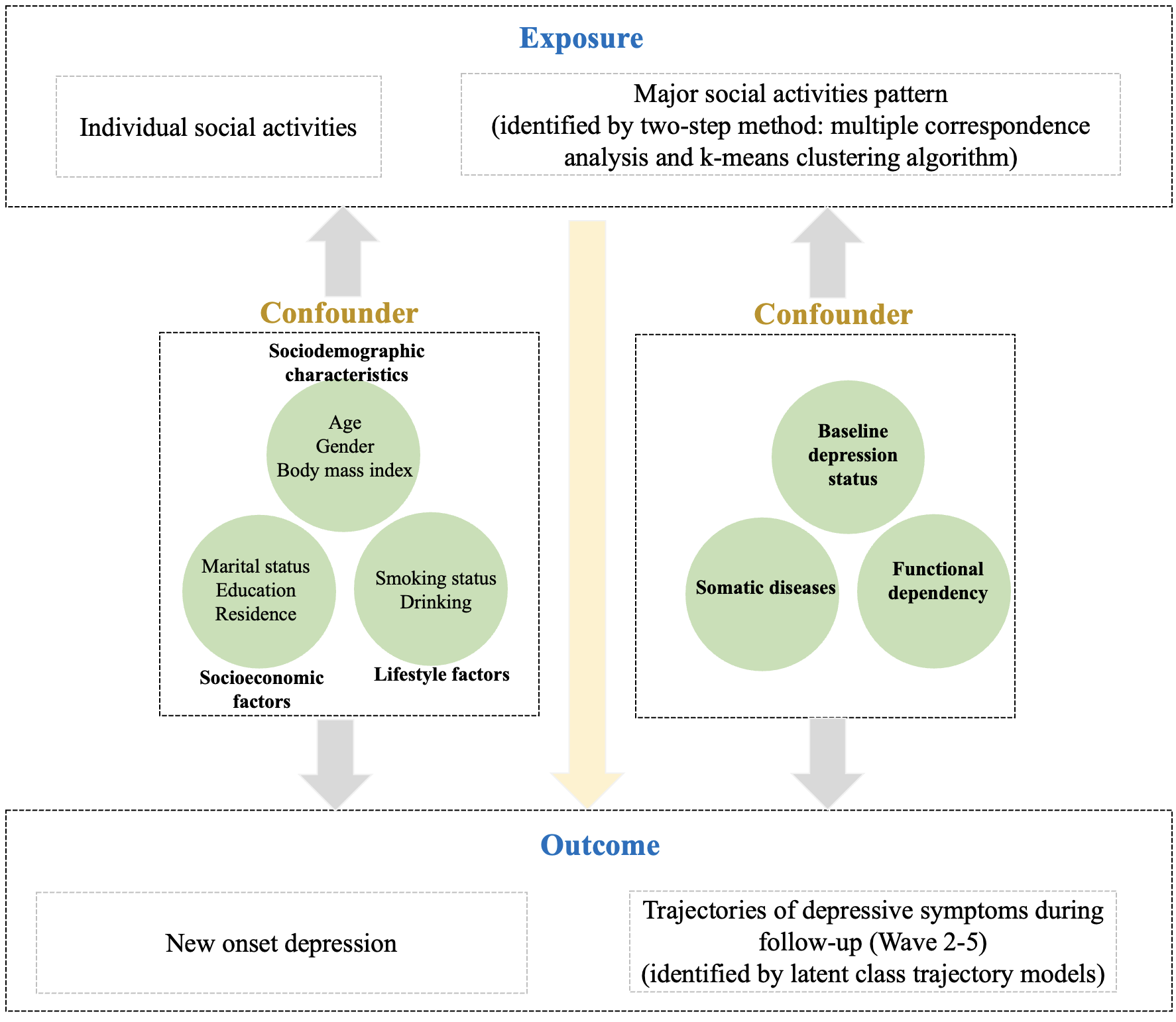
Figure S4. The Directed Acyclic Graph of the relationship between social participation, involved covariates, and depressive statuses among participants.

##
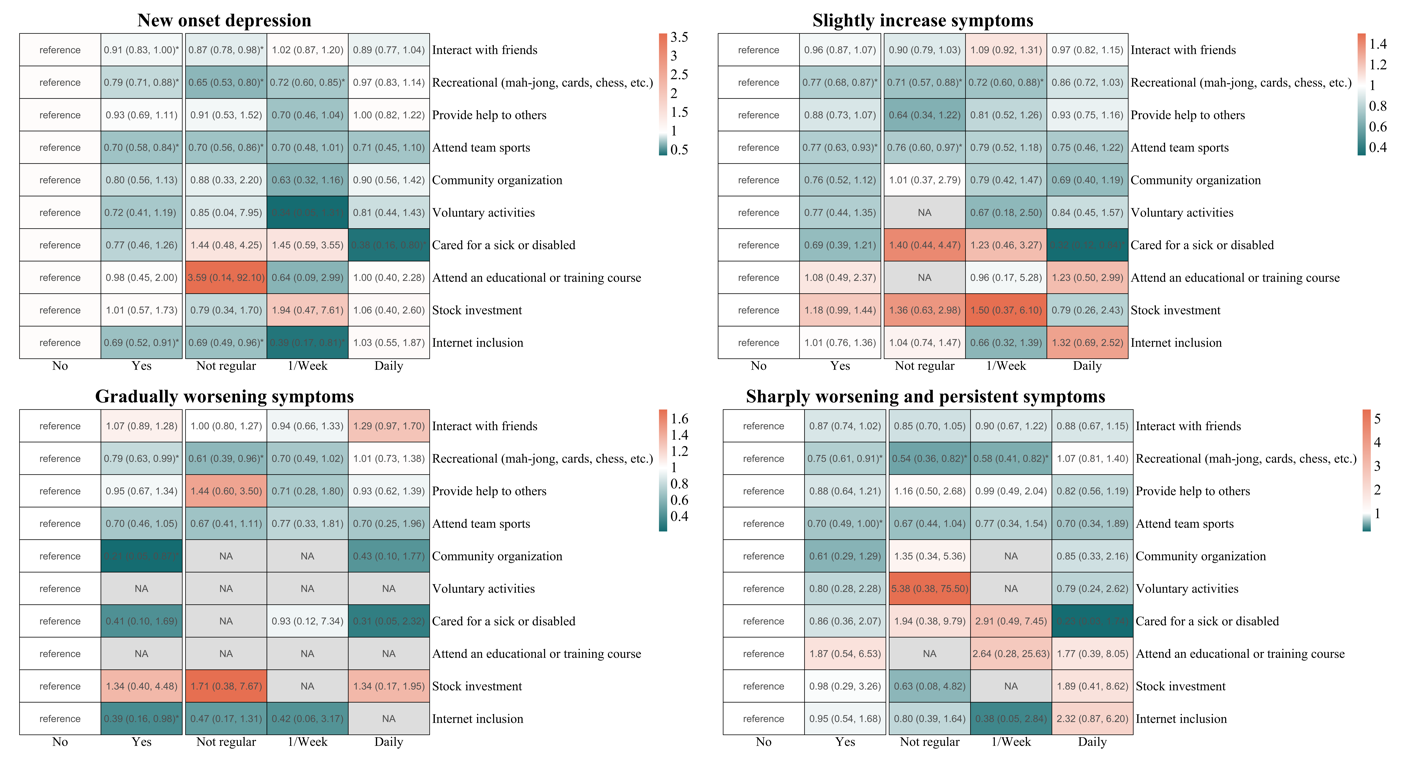
Figure S5. Risk of new onset depression and different trajectories of depressive symptoms over time among individuals with different social participation compared with that of individuals without such conditions by excluding patients missing baseline depressive status.

Multinomial logistic regression and logistic regression adjusted for confounding clarified by Directed Acyclic Graphs was used to estimate ORs and 95% CIs.

## Figure S6. Risk of new onset depression and different trajectories of depressive symptoms over time among individuals with different social participation pattern (identified by k-means) compared with that of individuals without such conditions by excluding patients missing baseline depression status.


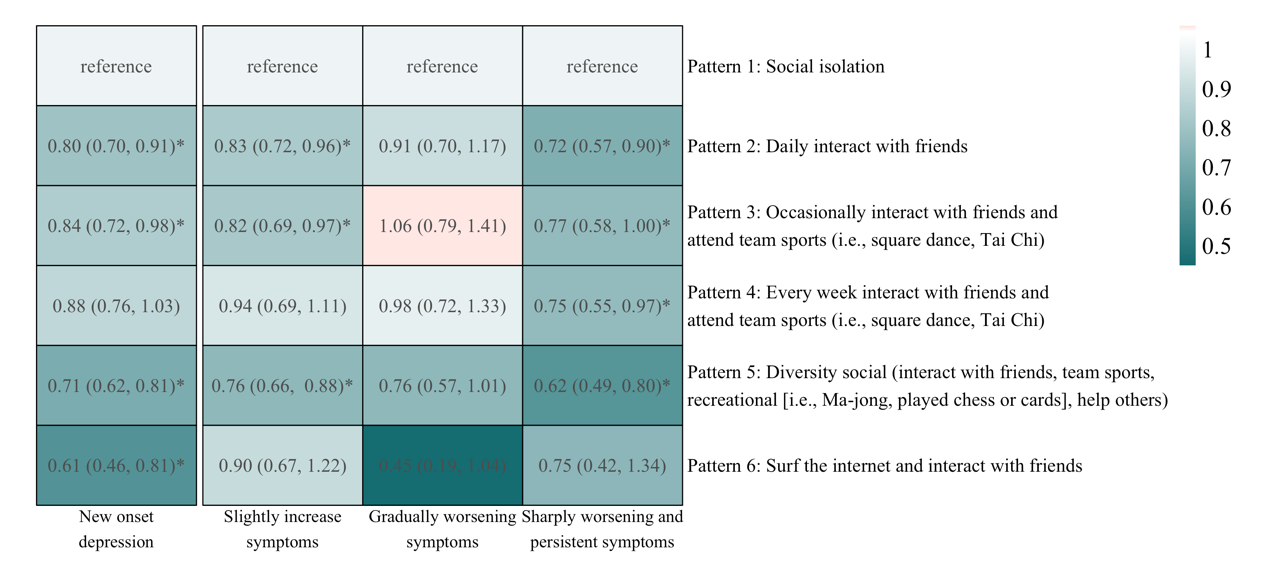


Multinomial logistic regression and logistic regression adjusted for confounding clarified by Directed Acyclic Graphs was used to estimate ORs and 95% CIs.
